# The COVID-19 pandemic in the African continent

**DOI:** 10.1186/s12916-022-02367-4

**Published:** 2022-05-02

**Authors:** Godfrey Bwire, Alex Riolexus Ario, Patricia Eyu, Felix Ocom, Joseph F. Wamala, Kwadwo A. Kusi, Latif Ndeketa, Kondwani C. Jambo, Rhoda K. Wanyenze, Ambrose O. Talisuna

**Affiliations:** 1 Department of Integrated Epidemiology Surveillance and Public Health Emergencies, Ministry of Health, P.O Box 7272, Kampala, Uganda; 2 School of Public Health, Makerere University, P.O. Box 7072, Kampala, Uganda; 3 Uganda National Institute of Public Health, Kampala, Uganda; 4 World Health Organization (WHO), Juba, South Sudan; 5 Noguchi Memorial Institute for Medical Research, College of Health Sciences, University of Ghana, Accra, Ghana; 6 Malawi-Liverpool-Wellcome Programme (MLW), Blantyre, Malawi; 7 Liverpool School of Tropical Medicine, Liverpool, UK; 8 Epidemic Preparedness and Response Cluster, World Health Organization, Regional Office for Africa, Brazzaville, Congo

**Keywords:** COVID, Africa, Pandemic, Infectious disease, Health, Epidemiology, Coronavirus, Vaccination, Coverage, Immunity, Essential health services

## Abstract

In December 2019, a new coronavirus, severe acute respiratory syndrome coronavirus-2 (SARS-CoV-2) and associated disease, coronavirus disease 2019 (COVID-19), was identified in China. This virus spread quickly and in March, 2020, it was declared a pandemic. Scientists predicted the worst scenario to occur in Africa since it was the least developed of the continents in terms of human development index, lagged behind others in achievement of the United Nations sustainable development goals (SDGs), has inadequate resources for provision of social services, and has many fragile states. In addition, there were relatively few research reporting findings on COVID-19 in Africa. On the contrary, the more developed countries reported higher disease incidences and mortality rates. However, for Africa, the earlier predictions and modelling into COVID-19 incidence and mortality did not fit into the reality. Therefore, the main objective of this forum is to bring together infectious diseases and public health experts to give an overview of COVID-19 in Africa and share their thoughts and opinions on why Africa behaved the way it did. Furthermore, the experts highlight what needs to be done to support Africa to consolidate the status quo and overcome the negative effects of COVID-19 so as to accelerate attainment of the SDGs.

## Introduction

### Dr. Godfrey Bwire

**Fig. 1:**
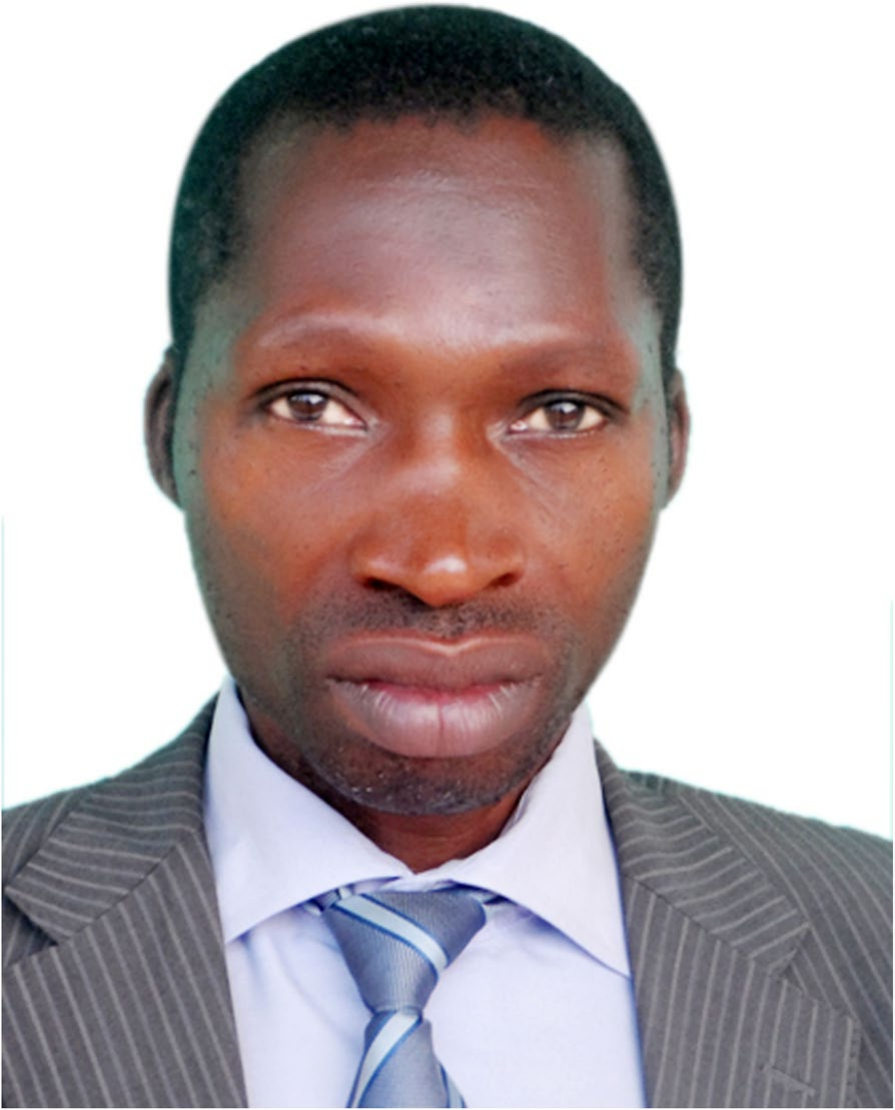
Dr. Godfrey Bwire (MBChB, MPH, PhD) is a medical doctor, researcher, and policymaker. Godfrey is the current head of the division of public health emergency preparedness and response of the Republic of Uganda Ministry of Health. In addition, Godfrey is a research fellow in the department of Community Health and Behavioral Sciences, School of Public Health, Makerere University, Kampala, Uganda. Godfrey’s research interests are on infectious diseases, public health, and cholera. Bwire et al. identified a new *Vibrio cholerae* transmission event from South Asia to East Africa, PMID: 29864113. Godfrey has an excellent peer-review record and has great interest in advancing research through, international collaborations, peer reviews, publications, and editorial work

Figure 1 shows the biography of Dr. Bwire.

In December 2019, new corona virus appeared in Wuhan, China [1, 2]. The new virus spread quickly, and in March 2020, the World Health Organization declared it a pandemic [3]. There was uncertainty and inadequate information about this new virus [2]. Soon, high-income countries such as the United States of America, Britain, Italy, Spain, and several others reported many cases and deaths and suffered from the negative effects of COVID-19 [4, 5]. Scientists and researchers predicted that low- and middle-income countries, such as those in Africa, would be the most negatively affected. Africa, being the least developed continent in terms of human development index [6], was to be the worst affected continent by COVID-19 infection with cases and deaths forecasted to overwhelming healthcare services [7]. In addition, Africa has lowest number COVID-19 research and least resources for public health. For instance, in some countries in Africa, under 10% of the population is vaccinated as compared to over 60% of the population in developed economies is vaccinated [8], yet Africa is the second largest and the second most populous continent with approximately 1.4 billion people (18% of the world population) in 2021 [9]. Africa’s low vaccination rate is a threat to global recovery from the effects of COVID-19 [10]. A significant proportion of the funding for COVID-19 in Africa has been by the individual governments which has the potential to divert resources from planned activities and consequently increase existing social disparities. Despite the underdevelopment and weaknesses in social services, the COVID-19 prediction and modelling were wrong on Africa and the continent has reported the least number cases and deaths of all the continents [11]. Other than the wrong COVID-19 prediction for Africa [12], there is uncertainty and inadequate research on COVID-19. Therefore, the main objective of this forum is to bring together infectious disease experts and researchers to give highlights of status of COVID-19 in Africa and to share their thoughts and opinions on why Africa behaved the way it did. In addition, this forum recommends ways and actions to support Africa to consolidate the status quo, overcome the negative effects of COVID-19, and accelerate attainment of Sustainable Development Goals by Africa.

The forum sections discuss the burden of COVID-19 in Africa giving reasons why the earlier predictions and modelling could not fit the reality. Dr. Godfrey Bwire elaborates on epidemiology of COVID-19 in African continent and explains why Africa has reported the lowest COVID-19 burden of all the continents. He notes that there is missing information on epidemiology of COVID-19 in Africa and recommends further research to fill this information gaps.

Next, Drs Alex Riolexus Ario and Felix Ocom and Patricia Eyu describe COVID-19 pandemic preparedness and responses in Africa. They highlight the interventions that helped African countries to successfully go through the various waves of the COVID-19 pandemic. The authors also discuss actions taken to control, mitigate, and respond to COVID-19 and how governments funded the COVID-19 response. Furthermore, Dr. Alex and the team highlight areas that need to be focused on to consolidate the current response, strengthen preparedness, and prevent future pandemics.

There are 54 independent States in Africa [13] all of which have reported COVID-19 cases at one time or the other [11]. There are also many states in Africa that are included on the World Bank list of fragile states [14]. The countries on the fragile states’ list grapple with poor social services due to conflicts, wars, internal migration, and displacement among other humanitarian crises [15]. There is little data on COVID-19 in Africa and less still when it comes to literature on COVID-19 pandemic in the fragile states. In this forum, Dr. Joseph Francis Wamala shares the lessons from the Republic of South Sudan on how this fragile state has been affected by COVID-19 amidst other humanitarian challenges. He deliberates on the epidemiology of COVID-19 in the Republic of South Sudan and points out areas that need to be addressed to support such fragile states to successfully deal with the COVID-19 pandemic.

In this article, Dr. Kwadwo Asamoah Kusi discusses COVID-19 seroepidemiology and effect of co-infections among cases reported in Africa. He notes that immune cross-reactivity between SARS-CoV-2 and other more common human coronaviruses has played an important role in distribution of COVID-19 infection in Africa. He also discusses the impact on COVID-19 prior exposure to common pathogens that are prevalent in Africa such as helminths, malaria, Tuberclosis, polio, and measles. He gives highlight on the clinical presentation of COVID-19 cases and reports that a significant proportion of SARS-CoV-2 infections have been asymptomatic and have therefore not been captured by health systems, and the weak laboratory testing capacity in Africa have not helped in detection of new cases.

On the other hand, vaccination using efficacious vaccines is one of the strategies fronted by WHO to combat COVID-19 [16–18]. However, inadequate availability of COVID-19 vaccines is a major challenge that shaped COVID-19 response globally and in Africa [19]. In this paper, Kondwani Jambo and Latif Ndeketa discuss the status of vaccination in Africa and give a comprehensive analysis of the barriers and enablers for vaccination in Africa. They also suggest ways to ensure stead vaccine supply and utilization in Africa.

In terms of impact to social services and economy, COVID-19 pandemic has impacted all sectors negatively [20, 21]. All countries have been affected regardless of the level of development. However, the effects have been more serious for the least developed countries where most of the African countries are placed. Professor Rhoda Wanyenze and Dr. Ambrose Otau Talisuna discuss the consequences of COVID-19 on the delivery of health services in Africa. They show the devastating impacts of COVID-19 in Africa and note that more is needed to support Africa to overcome the negative effects of the COVID-19 pandemic. They give proposals on actions that are needed to strengthen preparedness and to mitigate future pandemics in Africa.

**Competing interests**

Godfrey Bwire is an editorial board member for BMC Medicine.

**Authors’ contributions**

All authors were involved in drafting, editing, and revising the manuscript and agreed to its publication. All authors read and approved the final manuscript.

## Epidemiology of COVID-19 in Africa: why is COVID-19 incidence low in Africa?

### Godfrey Bwire

The coronavirus disease 2019 (COVID-19) was declared a public health emergency of international concern by the World Health Organization (WHO) on 30^th^ January 2020 [22], and on 11^th^ March 2020, it became a pandemic [23]. COVID-19 is the most destabilizing infection of the world to-date. It is also the second top documented infectious disease in the World coming after human immunodeficiency virus (HIV) [24]. COVID-19 is caused by a virus pathogen, severe acute respiratory syndrome coronavirus 2 (SARS-CoV-2). This viral infection has spread widely with unprecedented impact for social services and economies [25–27]. The infection found few countries globally prepared to mitigate its impact.

At the time when the WHO declared COVID-19 a global pandemic, scientists expected that Africa would be the worst affected in terms of incidence, prevalence, and mortality [28]. This prediction was unchallengeable due to the fact that Africa is the least developed of all the continents [29]. Moreover, Africa has a young population in which previous studies had shown to be more vulnerable during a pandemic due to a less-developed immune system against infectious diseases [20]. With time, this prediction passed and Africa reported the least number of cases and deaths compared to North America, Europe, South America, and Asia [30]. It is true that few studies and data are available on COVID-19 in Africa; however, the available studies are a true representation of COVID-19 status in the continent. As of 29^th^ December 2021, the reported cases out of Africa were the least at 3% (7,110,817/280,119,931) of the global COVID-19 cumulative total [31].

Like other emerging infections [32], controlling the spread of COVID-19 has been a big challenge to many countries of the world [33–35]. Thus, even with preparedness and prevention measures instituted, COVID-19 infection spread beyond Wuhan City and China [36].

**Fig. 2:**
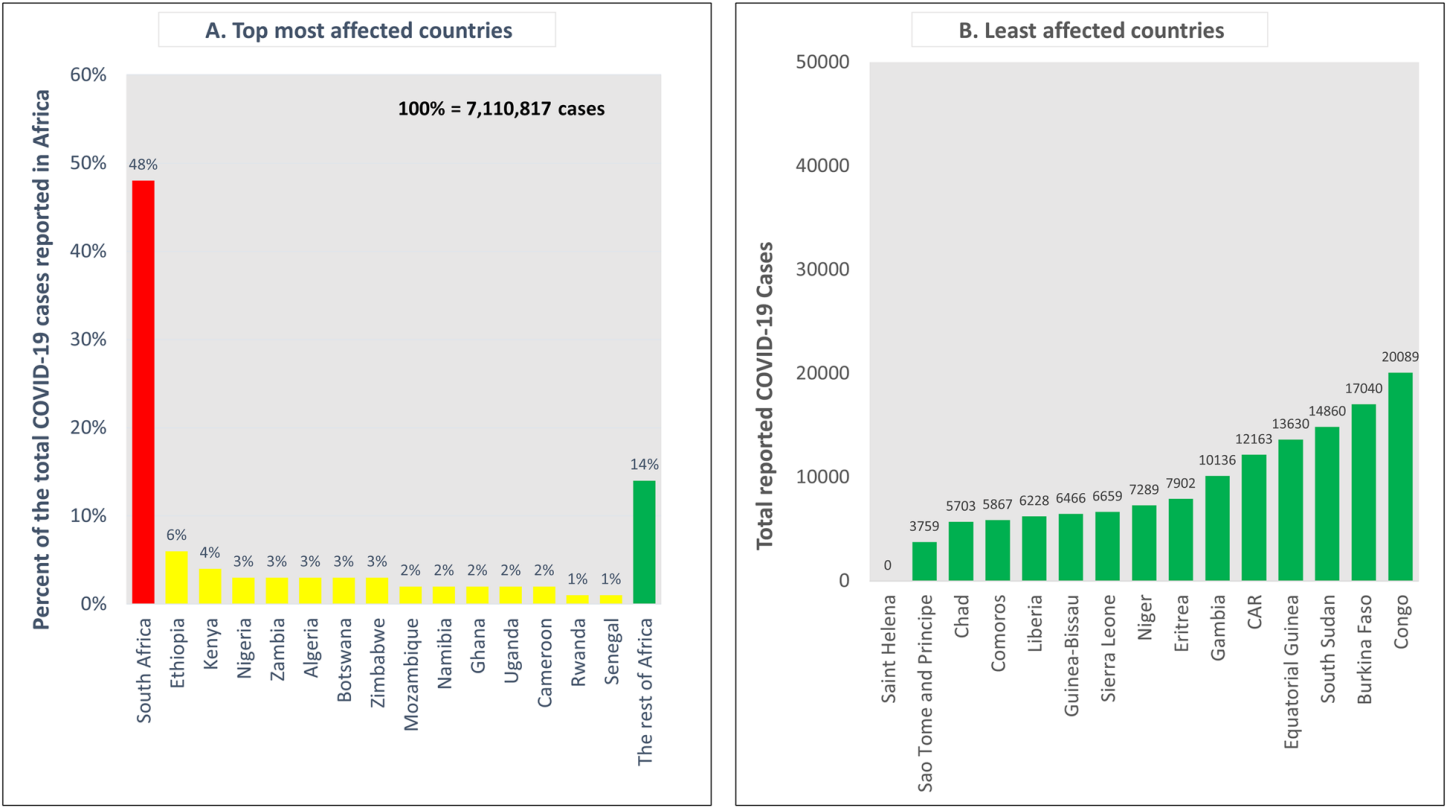
Shows the top and least COVID-19 affected countries in Africa after excluding the island states (which had very few cases) except Comoros. This analysis is based on COVID-19 data reported to the World Health Organization for the period 2020–2021. **A** The top most COVID-19 affected countries presented in the descending order**. B** Least affected countries in the ascending order after excluding the island states

According to literature, the first COVID-19 case in Africa was registered on 14^th^ February 2020 in Egypt [30]. Later, COVID-19 cases were reported by all other countries in Africa [37]. By the end of 2021 (29 December 2021), Africa had reported a cumulative of 7,110,817 cases and 155,505 deaths [37]. The majority of the cases (48%, 3,417,318/7,110,817) in the WHO African region were from a single country, the Republic of South Africa (RSA). If the island countries of Africa are excluded from the count due to the decimal number of cases from these states, the Republic of Chad becomes the least affected country on the mainland Africa with 5703 cases. However, the Republic of Chad reported more deaths than several other countries on the mainland WHO Africa region [37]. The most and the least COVID-19-affected African countries are shown in Fig. 2.

Countries with large economies and with most active travels reported the highest number of cases. Island countries, countries with a small landmass, and countries affected by conflicts reported the fewest number of cases. In the period 2020–2021, 86% (6,089,907/7,110,817) of all cases were from 15 countries with the rest of the continent (35 countries) contributing the 14% (1,020,910/7,110,817) of the reported cases.

In all these reports, COVID-19 pandemic was due to infection with the SARS-CoV-2 virus which evolved overtime. There were several variants of the original virus causing COVID-19 infections in Africa and globally [38]. Some of the notable variants that were associated with high infection rates, morbidity, and mortality were the beta (SARS-CoV-2 variant: B.1.351, delta (SARS-CoV-2 variant: B.1.617.2) and Omicron (SARS-CoV-2 variant: B.1.1.529.) [39–42].

**Fig. 3:**
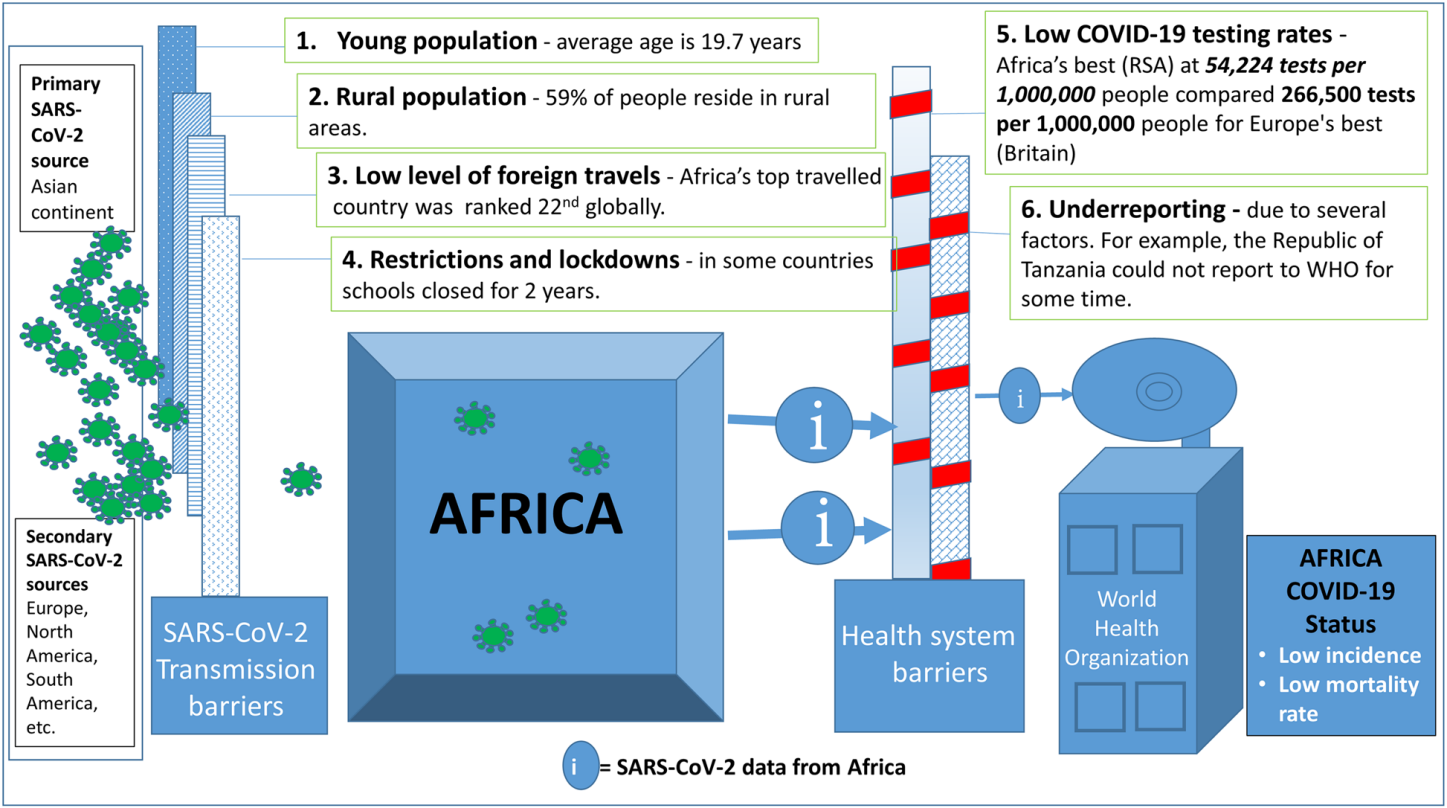
The barriers to COVID-19 introduction into Africa and reporting of cases and deaths to the World Health Organization. It is possible that the transmission / protective barriers to SARS-CoV-2 introductions and the health system barriers (low COVID-19 testing and low reporting of cases and deaths) could have complemented each other to result in low COVID-19 incidence and mortality for Africa during the period 2020–2021

There are various hypotheses to explain why Africa had the fewest number of COVID-19 cases compared to other continents, which is against the predicted trend. First, Africa has a young population where the average age is 19.7 years with 60% of the population being less than 25 years of age [43]. This young population has been found to be less susceptible to COVID-19 infection [44]. The second factor is that most (59%) of the people in Africa live in rural areas [45]. Rural people have limited travels and interactions with travellers / new communities that could be carrying the SARS-CoV-2 virus [46]. Third, low level of foreign travel in Africa compared to other continents [47]. The Republic of South Africa has the highest foreign travel in Africa and is ranked 22^nd^ globally [47]. This implies that the risk of acquiring travel-related infections such as COVID-19 for people in Africa is low. Fourth, countries in Africa moved very quickly to impose lockdowns and restrictions [48]. The early COVID-19 migratory restriction responses by the countries enhanced by leveraging on the existing infection control systems helped to reduce new infections [46]. Some countries took very drastic lockdown measures. The case in point is Uganda where schools were closed for 2 years [49]. Consequently, Uganda recorded the longest school closure of all the countries in the world. Lastly, due to limited testing capacity in Africa [50, 51], it is possible that the reported COVID-19 cases and deaths in Africa is a gross underestimation of the true disease burden on the continent. For example, Africa’s best facilitated country, the RSA, had carried out 54,224 tests per million population compared to Britain (Europe’s best) which carried out 266,500 tests per million population and for United States of America (North America) of 195,072 tests per million population in the same period [50]. Therefore, further studies are required on this topic to provide more accurate data. Furthermore, given the underdevelopment in Africa [52], it is possible that some infections and deaths have occurred but could not be reported. The possible factors responsible for the low COVID-19 incidence and mortality are summarized in Fig. 3.

There are other poorly understood factors related to COVID-19 incidence, mortality, and distribution within the African continent. For example, during the study period, December 2020 to December 2021, almost half (48%) of the COVID-19 cases reported to WHO from the African continent were from the Republic of South Africa. This high incidence and mortality of reported COVID-19 was also documented by other researchers whose study focused on epidemiology of severe COVID-19 in the Republic of South Africa [53]. There are several hypotheses put forward to explain this observation of the unexpectedly high reported COVID-19 cases by the Republic of South Africa [54]. One hypothesis is that of the population in the Republic of South Africa being at higher risk than the rest of Africa due the older median population age of 28 years [55] which is older age compared to the African average of 19.7 years [43]. However, if this was the case, then, the People’s Republic of Morocco with the median age of 29.1 years [55] would also report similar high number of COVID-19 cases. Another hypothesis for the Republic of South Africa’s higher reported cases is the higher incidence of comorbidities (communicable diseases mainly tuberculosis and human immunodefiency virus [HIV] and the non-communicable disease such as diabetes, obesity, and hypertension) [53, 55]. The third hypothesis for higher COVID-19 incidence in the Republic of South Africa could be due to better diagnostics and health care documentation that may allow for higher reporting rates [54]. In any case, the explanation for the higher numbers by the Republic of South Africa is still not clear. Therefore, additional in-depth studies are required to test the various hypotheses and to give better understanding of why the Republic of South Africa reported such a high proportion of COVID-19 incidence and mortality.

In conclusion, though the number of COVID-19 cases and deaths reported in Africa were low, there is a lot that is yet to be understood. For instance, the data reported are not segregated to understand sex/gender differences. Also, some few countries such as the United Republic of Tanzania did not accept COVID-19 as a problem initially. Hence, it would be interesting to know how this affected the trend of the pandemic in the communities therein. Furthermore, in order to build a resilient global community, countries in African will need to be supported by the more developed countries to overcome the current health and development issues. Currently, a number of countries get support from the developed countries [56, 57]; however, this support is inadequate and sometimes misdirected [58–61]. To ensure that the current health and development issues in Africa are sustainably addressed, more focused funding support will need to be employed. 

## COVID-19 pandemic response, preparedness and prevention in Africa

### Alex Riolexus Ario, Patricia Eyu, and Felix Ocom

**Fig. 4:**
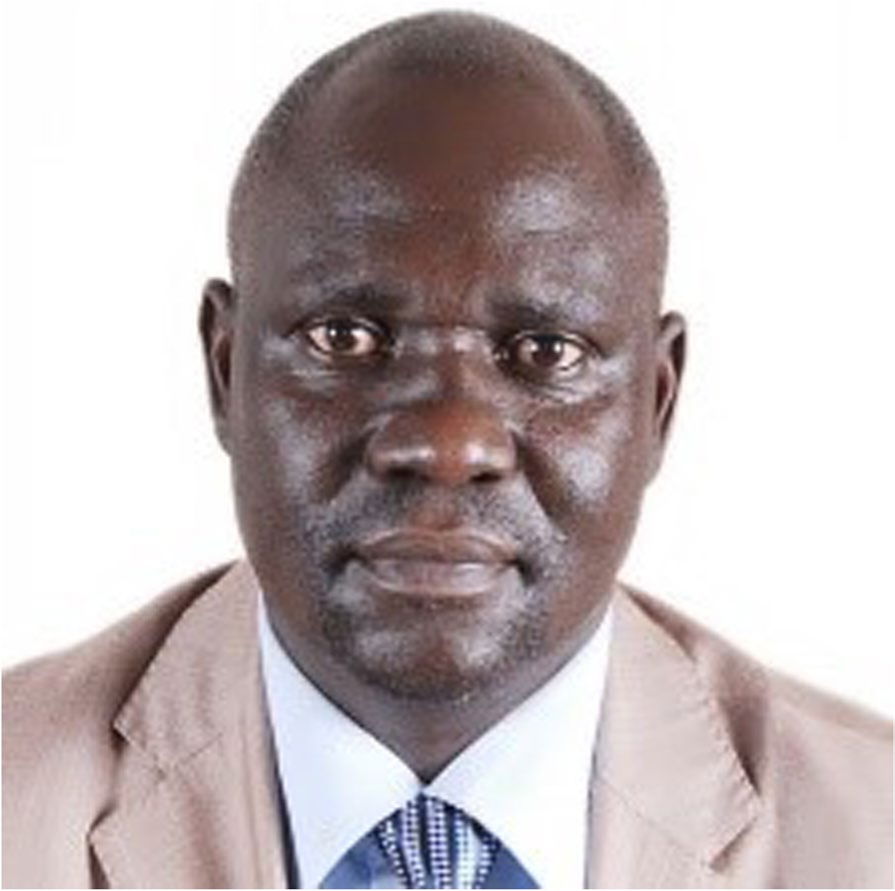
Alex Riolexus Ario is the Director of Uganda National Institute of Public Health. A Public Health Specialist with extensive knowledge in epidemiology, health policy, health systems, and quality improvement models. He has worked in various capacities in Ministry of Health and served as a member in numerous TWGs, Steering Committees, and Boards; Board Member, Africa CDC’s Journal of Public Health in Africa; Member, Africa Mortality Surveillance Taskforce, Advisory Committee, Koffi Annan Global Health Leadership Program and Africa COVID-19 Surveillance Taskforce; Deputy Chair, East Africa Mortality Surveillance Taskforce and Uganda COVID-19 Inter-Agency Technical Taskforce; Chair East-Africa Regional Technical Advisory Committee and IANPHI, Africa Network

Figure 4 shows the biography of Dr. Ario.

**Fig. 5:**
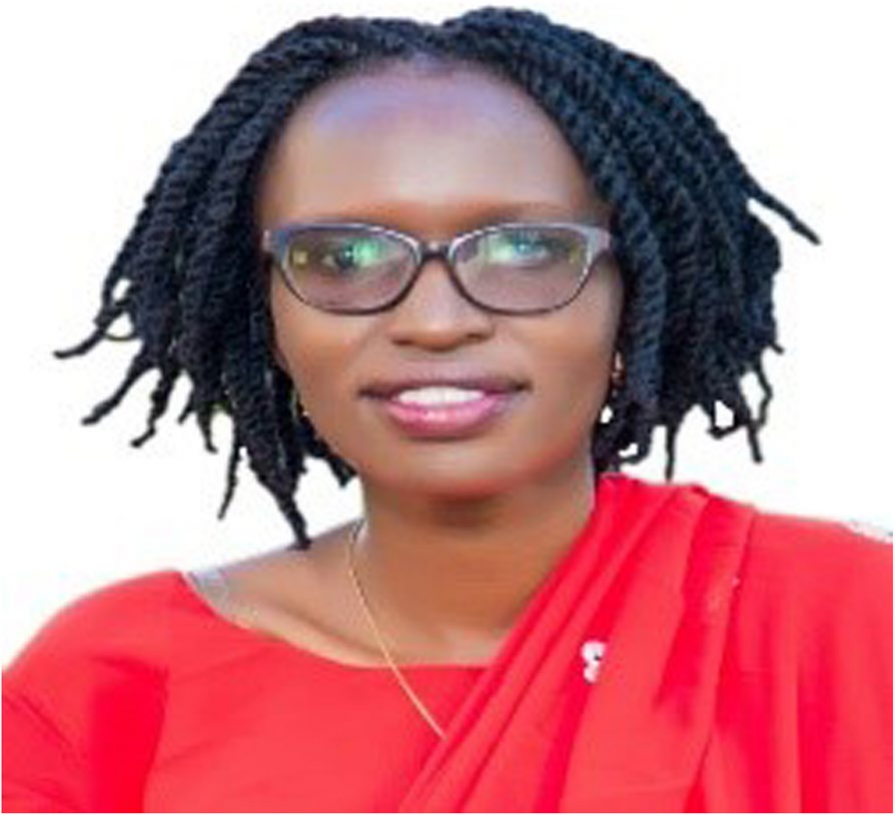
Eyu Patricia, BBLT, MPH, is a field epidemiologist with 4 years of experience in public health and epidemiology. She has worked in both private and public institutions and supported the Ministry of Health under various positions. Most recently, Patricia supported the Uganda COVID-19 response in case investigation, strengthening the surveillance structures at the districts, alert reporting, and developing surveillance tools. She has publications in food poisoning and other disease outbreak investigations. Patricia is currently a field epidemiology trainer in the Field Epidemiology Training Program-Intermediate level and part of the mentorship team in the Advanced FETP

Figure 5 shows the biography of Ms. Patricia Eyu.

**Fig. 6:**
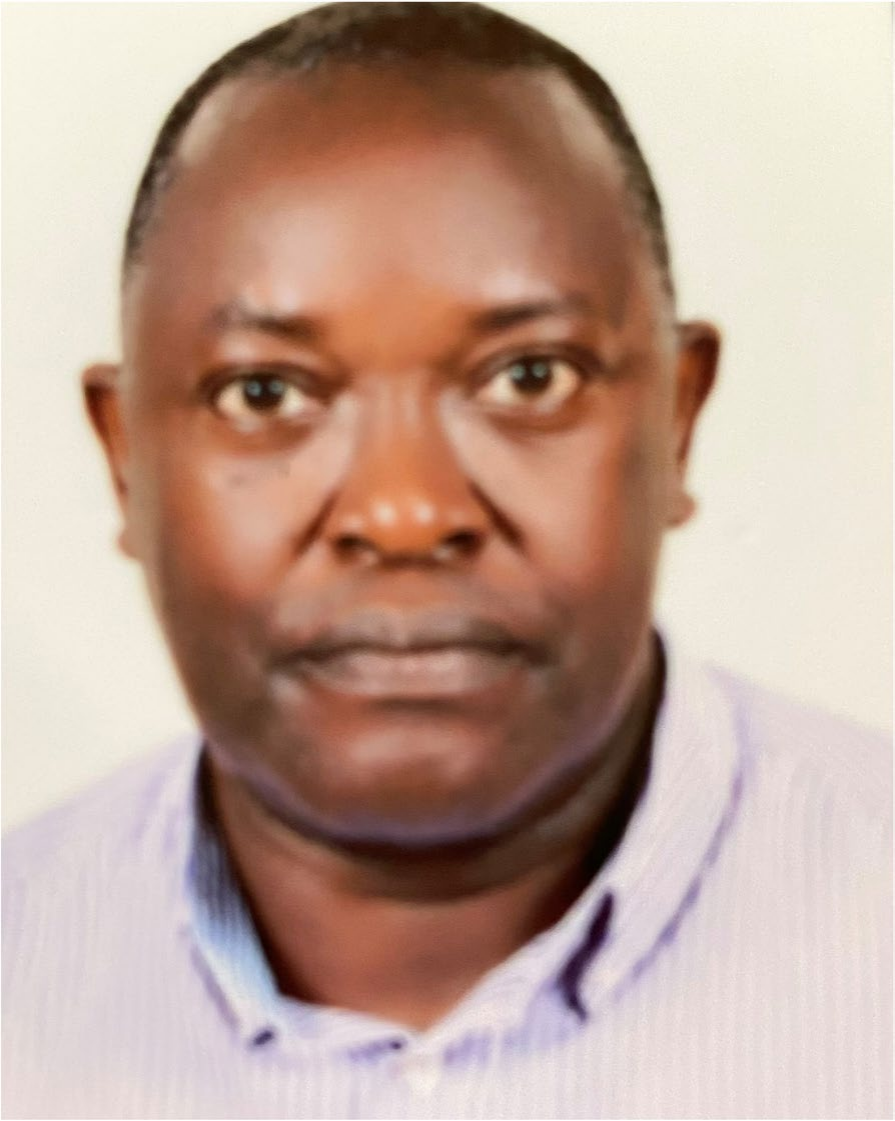
Felix Ocom is the Deputy Manager of the National Public Health Emergency Operations Centre, Ministry of Health, Uganda, and a member of COVID-19 Incident Management Team. Felix is a national trainer for the Uganda National Rapid Response Teams for public health emergencies, and Integrated Disease Surveillance and Response. He participates in the development, implementation, and evaluation of national public health emergency preparedness and response strategies, in line with International Health Regulations (2005). Felix has worked with the WHO (2016–2020) as a consultant epidemiologist, US CDC (2008–2012) as a public health specialist (HIV Biomedical Prevention), and in various positions in the public health sector in the Government of Uganda. Felix’s interests include public health emergency management and building resilient systems for public health emergency preparedness and response at community levels

Figure 6 shows the biography of Dr. Ocom.

Following the declaration of COVID-19 as a Public Health Emergency of International Concern (PHEIC) and pandemic on 30 January, 2020 [22, 62], the World Health Organization (WHO) issued several recommendations that were adopted by different countries to prevent spread of COVID-19 [63, 64]. The non-pharmaceutical recommendations included physical distancing, wearing a mask, keeping rooms well ventilated, avoiding crowds, and cleaning hands with alcohol-based hand rub or soap and water [63, 65]. Multiple communication platforms ranging from social media, radio, and messaging were used to conduct mass health education and sensitization of the public on COVID-19 [66, 67].

The governments of several African countries including Uganda, Kenya, Malawi, and Ghana implemented a series of vulnerability reduction and containment measures to curtail transmission of COVID-19, and these included closure of international airports; closing ground crossing points for passengers with the exception of cargo drivers; closure of schools and other high congregation points; freezing of public and private transport; outlawing all mass gathering events; overnight curfew; and nationwide lockdowns [68–71]. Contact tracing, quarantine, and isolation of confirmed COVID-19 cases were also implemented to minimize the spread of infection in South Africa, Uganda, Rwanda, and Nigeria [66, 71, 72].

In Uganda, the Ministry of Health developed the COVID-19 preparedness and response plan to provide a framework for coordination and control of COVID-19 in the country [67]. Similar preparedness and response plans were crafted in Ghana, Malawi, and Kenya to address the COVID-19 threat [68–70]. National task forces (NTFs), incident management teams, district task forces, and their sub-committees were activated as the center for coordination in various African countries [67–70].

The NTF sub-committees were for effective coordination and management of interventions as each sub-committee had specific terms of reference. In Kenya, for example, five technical sub-committees were developed which include coordination; surveillance and laboratory; case management and infection prevention and control; risk communication; and logistics [69]. Other African countries had similar sub-committees with Malawi including partner coordination; and Uganda continuity of essential services and strategic information, research, and innovation [67, 70].

While most of the countries agreed with WHO on COVID-19 prevention, there were some few countries which thought otherwise. For instance, in Tanzania, a COVID-19 response plan was developed between January and June 2020 [73]. However, by June 2020, the country was declared COVID-19 free and prayers were advised as a remedy [74].

At the start of COVID-19 pandemic, there was no laboratory capacity to detect SARS-COV-2 in Africa. However, in early March, just over a month later, 42 African countries had developed the expertise and resources to perform COVID-19 testing in both public and private laboratories. Uganda, Nigeria, and Kenya opened up airports and land borders, screening of travellers was emphasized, and to-date, one has to have a negative polymerase chain reaction test result for COVID-19 for entry clearance into another country [75–77].

Uganda and the Republic of South Africa adopted an online results dispatch system for reporting of COVID-19 results where an individual who has access can easily download and print results and in other instances WhatsApp messages of results were sent directly to the individual who was tested [78, 79]. 

In Uganda, investors, private sector players, institutions, and individuals contributed towards financing the country’s response to the COVID-19 pandemic [80]. Funding support to manage the COVID-19 pandemic in some African Countries was also obtained from various partners like the World Health Organization, African Development Bank, UNICEF, The Global Fund, The World Bank, Africa Centers for Disease Control and Prevention, US Centers for Disease Control and Prevention and Direct Relief among others [81–87].

With all the aforementioned interventions that were put in place to curb the pandemic, some of the measures did not go well in some countries. In Uganda, for example, schools were closed when learners were already exposed to COVID-19 so they took the infection to their families contributing to widespread community transmission in the country [88]. There was also a lot of stigma in the communities so persons who tested positive for COVID-19 feared to identify themselves for isolation [88]. In Kenya, some funds intended to support the vulnerable poor was diverted for unintended use and health workers did not have enough protective equipment to protect them from COVID-19 [89, 90]. In South Africa, lockdown was successfully implemented but it did not have intended impact of reducing the number of cases [91].

African countries developed multi-sectoral plans which ensured preparedness and timely, consistent, and coordinated response to COVID-19 pandemic. Engagement of partners, including the private sector, was critical in the response efforts. Strengthening surveillance systems, increasing the number of responders including epidemiologists and community health workers, improving hospital infection prevention and control, improving diagnostic testing capabilities, strengthening critical care, and revising public health legislature to fast-track authorizations were crucial in COVID-19 response in Africa.

**Competing interests**

The authors declare that they have no competing interests.

**Authors’ contributions**

ARA led the writing process after collecting program data, analyzed and interpreted the data, coordinated manuscript writing, and wrote the first draft; ARA and PE participated in data collection, analysis, writing, and revision; all authors revised the manuscript draft critically for key intellectual content and read and approved the final manuscript.

## COVID-19 situation and management in conflict-affected states: a case of South Sudan

### Joseph F Wamala

**Fig. 7:**
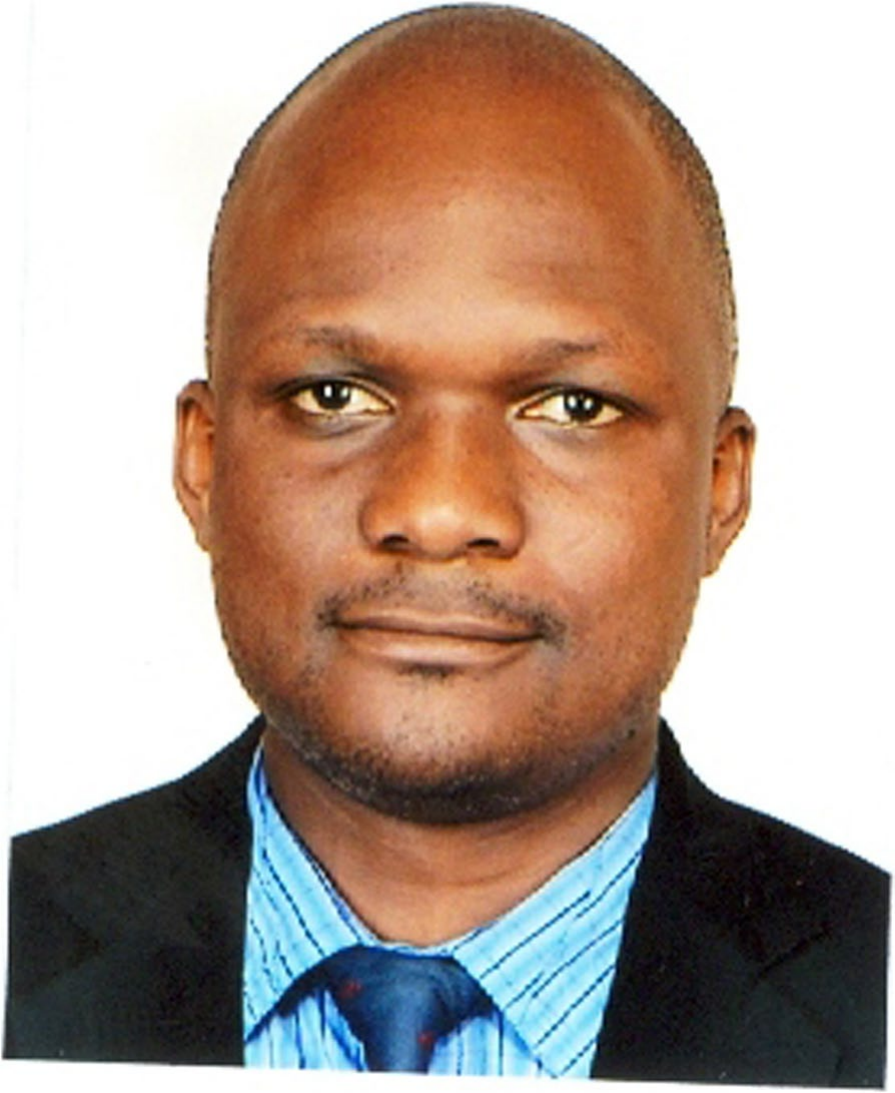
Dr. Wamala Joseph Francis is the Country Preparedness Officer in the WHO Country Office for South Sudan, Juba. He holds a Medical Degree and a Master’s in Public Health from Makerere University, Kampala, Uganda and a Ph.D. in Public Health Epidemiology from Walden University, Minnesota, USA. His professional career and research work focuses on public health surveillance and epidemiology of epidemic and pandemic prone diseases in the developing and humanitarian contexts. Dr Wamala has over 20 years of work with national Ministries of Heath, one-health stakeholders, and other actors critical for strengthening emergency preparedness, response readiness, and public health security

Figure 7 shows the biography of Dr. Wamala.

South Sudan is faced with a protracted humanitarian crisis that is compounded by several shocks including displacement, severe food insecurity, protracted flooding, and COVID-19 [92, 93]. As a result, South Sudan currently hosts over 2 million internally displaced persons and an estimated 329,000 refugees [92]. These displaced populations are vulnerable and have faced several outbreaks in the past including cholera, hepatitis E, malaria, and measles [94–96]. These outbreaks have been precipitated by inadequate physical access to essential health services currently estimated at 44%, and insufficient access to safe drinking water and basic sanitation estimated respectively at 41 and 16% [97]. The water, sanitation, and hygiene (WASH) indicators are even lower in displaced populations, with recent hepatitis E outbreaks attributed to WASH indicators that are below the sphere threshold [98]. These WASH conditions are rife for COVID-19 transmission in displaced populations. Recent surveys in Bentiu camp have revealed significant gaps in access to handwashing points, soap, and water containers [98]. Moreover, the risk of adverse COVID-19 outcomes in displaced populations is likely to be accentuated by comorbidities like malnutrition, tuberculosis, and human immunodeficiency virus (HIV) [99]. South Sudan is also faced with threats of importing outbreaks like Ebolavirus from other countries in the region [100].

Hence in 2019, the South Sudan government, with support from partners including the United Nations Agencies, International and national Non-Governmental Organizations (NGOs) embarked on preparedness efforts to strengthen disease surveillance, molecular testing, and case management capacities [100]. The capacities and lessons learnt from these preparedness efforts came in handy to support effective and timely initial and subsequent response to COVID-19 [101]. South Sudan has a nascent laboratory network with SARS-CoV-2 Nucleic Acid Amplification capacities largely concentrated at the national level and in two sub-national laboratories. Consequently, most of the other sub-national locations including displaced populations leverage the National Tuberculosis program four-module GeneXpert platforms to support COVID-19 testing. The other sites rely solely on Antigen rapid diagnostic testing (Ag-RDT) for SARS-CoV-2 testing in suspect or probable COVID-19 cases. Hence by 21 December 2021, South Sudan had performed 287,890 SARS-CoV-2 tests. This translates into 2.60 tests per 10,000 per week, which is far below the testing threshold of 10 tests per 10,000 per week [102]. These statistics highlight the inadequate testing capacities and reduced propensity to accurately quantify COVID-19 caseloads in the displaced communities.

A COVID-19 serosurvey in Juba showed that at least 103 cases were unreported at the community level for every polymerase chain reaction (PCR) confirmed case [103]. These findings highlight the possibility of unreported community transmission given the current surveillance and testing gaps in the country, which are even worse in displaced populations. As a consequence, a substantial number of suspect COVID-19 cases may not be tested, leading to undetected community transmission. The testing gaps also affect follow-up interventions like case isolation, contact identification, and quarantine, thus compromising the quality of the response with a risk of adverse COVID-19 outcomes in vulnerable populations like displaced people [104].

The South Sudan COVID-19 response is led by the National Taskforce and National Steering Committees, which provide policy and technical oversight to the overall response, guided by the national strategic preparedness and response plan and relevant guidelines [101, 104]. Critical to the South Sudan COVID-19 response is the work involving community networks and influencers for effective communication and community engagement directed to addressing information needs for interrupting COVID-19 transmission [101].

Various countries have implemented population wide restrictions to delay transmission peaks and protect health services [105]. However, these restrictions are associated with negative social and economic consequences that worsen preexisting vulnerabilities in displaced populations [106]. In this regard therefore, South Sudan implemented time-limited partial lockdowns that included restrictions on social, religious, and cultural gatherings, community-led shielding of vulnerable populations through vaccination with COVID-19 vaccines, home isolation of the mild to moderately ill COVID-19 cases, and facility-based management of severe and critically ill cases in designated treatment facilities [107].

The quantification of COVID-19 vaccines for displaced populations is currently incorporated in the South Sudan COVID-19 vaccine deployment plan [108]. South Sudan displaced populations (IDPs and refugees) are estimated to be 3.2% of the total population and are included in the initial prioritized 20% of the national population to be vaccinated with COVID-19 vaccine [108]. The other prioritized populations include health workers, the elderly (65 years and above), persons with co-morbidities, persons living with HIV/AIDS, teachers, and other essential workers with high risk of infection [108]. However, South Sudan is one of the countries in Africa that have not been able to attain the COVID-19 vaccine coverage target of reaching 10% of the population by September 2021and 40% of the population by December 2021 respectively [109]. The low COVID-19 vaccine coverage in South Sudan is attributed to low vaccine uptake [107, 110]. Following a WHO support mission to South Sudan, a new strategy has been developed to accelerate COVID-19 vaccine uptake [110]. The strategies proposed include static and outreach vaccinations targeting high risk and congregate locations like urban centers and displaced populations as well as cross-border populations and other strategies tailored to the local context [110].

Overall, South Sudan continues to brace the impact of a protracted humanitarian crisis that has accentuated vulnerabilities and eroded coping capacities across all sectors including basic health and emergency response. The ongoing COVID-19 response in the displaced populations and the country at large should reinforce the whole-of-society engagement of stakeholders and communities to optimize surveillance, testing, COVID-19 vaccine uptake, adherence to public health social measures, communication and community engagement, provision of appropriate clinical care, and sustain essential health and social services.

**Competing interests**

The author declares no competing interests.

**Authors’ contributions**

All authors were involved in drafting, editing, and revising the manuscript and agreed to its publication. All authors read and approved the final manuscript.

## COVID-19 seroepidemiology in Africa and the effect of co-infections—an immunological perspective

### Kwadwo A Kusi

**Fig. 8:**
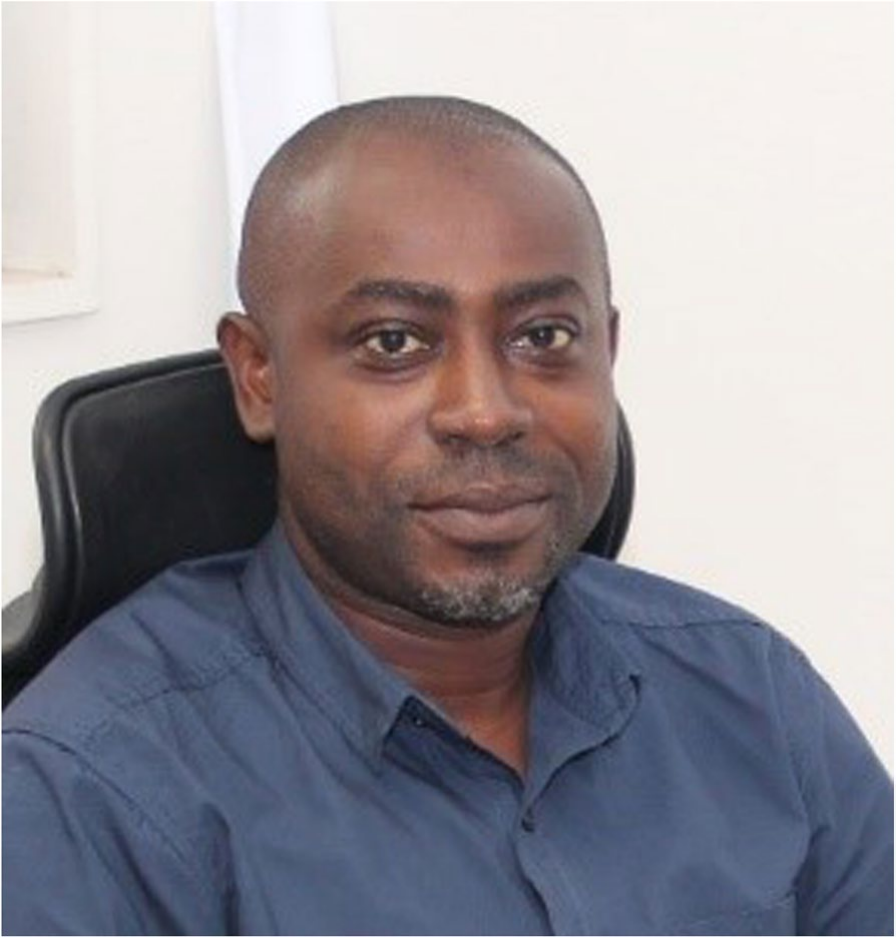
Dr. Kwadwo Asamoah Kusi is a Senior Research Fellow in Immunology at Noguchi Memorial Institute for Medical Research (NMIMR), University of Ghana. He holds BSc and MPhil degrees in Biochemistry from University of Ghana and a PhD in Medicine from Leiden University Medical Centre, The Netherlands. His research focuses on identifying protective immune response targets in *Plasmodium* and on multi-allele vaccine formulation strategies to counter the strain-dependent effects of antigen polymorphism on immune responses. Dr. Kusi is lead immunologist on ongoing clinical trials at NMIMR. He was recipient of the Ghana National Youth Achievers Gold Award for Science in 2012

Figure 8 shows the biography of Dr. Kusi.

The SARS-CoV-2 virus infects host cells using its spike (S) protein to interact with the host cell angiotensin-converting enzyme 2 (ACE2) receptor [111]. The pathophysiology of COVID-19 infection is due to both the humoral (antibody mediated) and cellular immune (T-lymphocyte and innate cell mediated) responses, although the underlying mechanisms are just beginning to be dissected. The disease spectrum ranges from asymptomatic viral infection, through mild and moderate symptoms but can quickly progress to severe disease with complications. During the asymptomatic to mildly symptomatic phases of the disease, the immune responses is largely normal as expected, with significant neutralizing antibodies and highly activated T cells [112]. Severely sick COVID-19 patients however tend to have an infection-related disproportionate increase in the numbers of innate cells such as neutrophils, monocytes, and macrophages, relative to the number of lymphocytes [113, 114]. The non-specific immune responses, mostly from innate immune cells, are therefore more likely to be associated with the observed immunopathology. Highly activated cells of the innate immune system, including macrophages, neutrophils, and dendritic cells, have been shown to predominate in lung tissues of COVID-19 patients [113]. Dendritic cells and macrophages express toll-like receptors that are used in sensing viral RNA and lead to the activation of the nuclear factor kappa B (NF-κB) pathway and the subsequent induction of pro-inflammatory cytokines. Excessive production of cytokines such as interleukin-1 beta (IL-1β), IL-6, and tumor necrosis factor-alpha (TNF-α) has been shown to result in a virus-induced hyperinflammatory condition known as the cytokine storm, which is associated with severe COVID-19 complications and an aggravation of lung pathology [115].

Aside these pathology-associated immune responses, specific adaptive responses to viral antigens have been demonstrated, although the extent of protection attributable to these responses remains to be fully established. Aside from immunity, other factors such as age, gender and comorbidities [116], race [117], socioeconomic status [118], and infectious disease burden [119] have variously been identified as important factors that predispose infected persons to either severe, fatal disease, or asymptomatic/mild disease. The cell-mediated immune response is the principal and effective immune response against viral infection. However, humoral immunity is an important complement that is required for effective response [120]. The antibody response is greatest against two main proteins; the spike protein located on the viral surface and hence of significant protective value, and the nucleocapsid protein which is intracellular [121]. Virus-neutralizing antibodies and long-lived memory B cells are however mostly against the receptor binding domain of the spike protein and are more prevalent in severe COVID-19 patients rather than those who have mild or asymptomatic infections or have recovered [122, 123]. Antibodies against other human corona viruses (hCoVs) that show cross-reactivity with SARS-CoV-2 antigens have also been described [124, 125] and may give an indication of some benefit of previous exposure to these other hCoVs for protection against COVID-19. While neutralizing antibodies have been shown to be highly effective against the cognate viral variants, heterologous variants such as delta and beta do show some level of immune escape [126], and there are early indications that the omicron variant may have an even greater immune escape capacity, judging from the number of mutations in its essential proteins.

In addition to antibodies, T cells have also been shown to be highly important for SARS-CoV-2 immunity. The specific T cell effector and memory response is critical to sustaining good and hopefully protective immunity. CD4+ T cells have been shown to target several of the viral proteins, while CD8+ T cells are mostly against the spike protein and nucleoprotein especially [127]. Again, T cell epitopes that are cross-reactive between SARS-CoV-2 and other hCoVs have been identified [128], re-emphasizing the potential of cross immunity to SARS-CoV-2 in persons with prior exposure to other hCoVs. Indeed, antigen-specific sequence alignments for a number of the essential SARS-CoV-2 antigens from different hCoVs show significant homology [129].

A significant proportion of SARS-CoV-2 infections have been asymptomatic and have therefore not been captured by health systems. This, combined with the low levels of SARS-CoV-2 testing in Africa has made seroprevalence studies with specific reagents a better proxy of the extent of natural infection [130]. These SARS-CoV-2-specific antibody seroprevalence studies confirm the fact that an overwhelming proportion of infections in Africa go undetected. Despite their importance, differences in detection kits can complicate comparison of seroprevalence data from different parts of the continent. Nwosu et al. [131] reported an antibody seroprevalence of up to 30% from sampling over 900 persons in Yaounde district, Cameroon, and this was more than 300 times the reported national case count from PCR testing. A study in Ghana with rapid kit detection of anti-nucleocapsid protein IgG and IgM reported up to 27% antibody seropositivity in populated areas such as lorry stations and markets, as compared to 10% seropositivity in shopping malls mostly patronized by the affluent [132], and these are much more than the reported infection rate from national case counts. A study among healthcare workers in Kenya found up to 20% IgG seropositivity against the SARS-CoV-2 spike protein in highly populous regions and about half that level of seropositivity in less populated areas [133]. A survey among Kenyan blood donors in multiple counties also revealed an average to 5% seropositivity, with higher rates of up to 8% in urban counties [134]. Even higher seroprevalence rates have been described among blood donors from Malawi, with up to 80% of donors in urban areas being seropositive during peak transmission periods [135]. While these data present a better picture of the extent of exposure to infection, they may sometimes be difficult to compare across different countries and regions because of the differences in antibody measurement approaches.

Aside from cross-reactivity among hCoVs, there is also growing evidence of immune cross-reactivity between SARS-CoV-2 and other more common human corona viruses [136, 137]. Cross-reactive antibody and T cell responses against the spike and nucleoproteins especially have been described [138, 139].

Immunity to COVID-19 could also be impacted by prior exposure to common pathogens such as helminths, malaria, TB, polio, and measles, most of which are prevalent in most parts of sub-Saharan Africa [140]. Despite most of these diseases being killers by themselves, the high levels of exposure to the associated pathogens does impact the immune environment in persons who survive infection. First, persons living in high malaria transmission areas who have more frequent parasite exposures have been shown to develop a higher tolerance to inflammation and/or can tolerate higher parasite burden compared to persons who live in low transmission areas [141, 142]. This phenomenon of the induction of immunological tolerance has also been described for other pathogens, including helminths, bacteria, and viruses [143–145]. The infection-experienced host is therefore able to tolerate SARS-CoV-2-induced inflammation and does not suffer significant COVID-19-mediated immunopathology.

Secondly, there is growing evidence that the innate immune response against pathogens can develop a memory phenotype which can be recalled following subsequent infection with other, different pathogens [146]. This phenomenon, called trained immunity, is believed to cross-protect against SARS-CoV-2 infections. There is some evidence of COVID-19 cross-protection in persons who have taken the live measles, polio, and BCG vaccines [147–149], and this immunological cross-talk may also occur with natural infection with the respective pathogens. HIV/AIDS may be an exception to this phenomenon because it is not just an infection that modulates the immune system but also causes immunosuppression.

In summary, the immune response to SARS-CoV-2 can be associated with both disease pathology and protection against disease. Management of COVID-19 symptoms and complications therefore requires a careful control of pathology-associated immune and inflammatory responses, and a focused enhancement of protection-associated responses. The former has been attempted with the use of immunosuppressive therapies for managing severe COVID-19 symptoms while the latter will be fulfilled by appropriate vaccine design, which now needs to be updated to cover emerging variants of concern. A better understanding of the interaction between SARS-CoV-2 and other infections will also be required to decipher protective immune mechanisms and for the management of COVID-19 in areas with a high burden of infectious diseases.

**Competing interests**

Kwadwo Kusi is an editorial board member for BMC Medicine.

**Authors’ contributions**

All authors were involved in drafting, editing, and revising the manuscript and agreed to its publication. All authors read and approved the final manuscript.

## COVID-19 vaccination uptake and coverage in Africa

### Latif Ndeketa and Kondwani C. Jambo

**Fig. 9:**
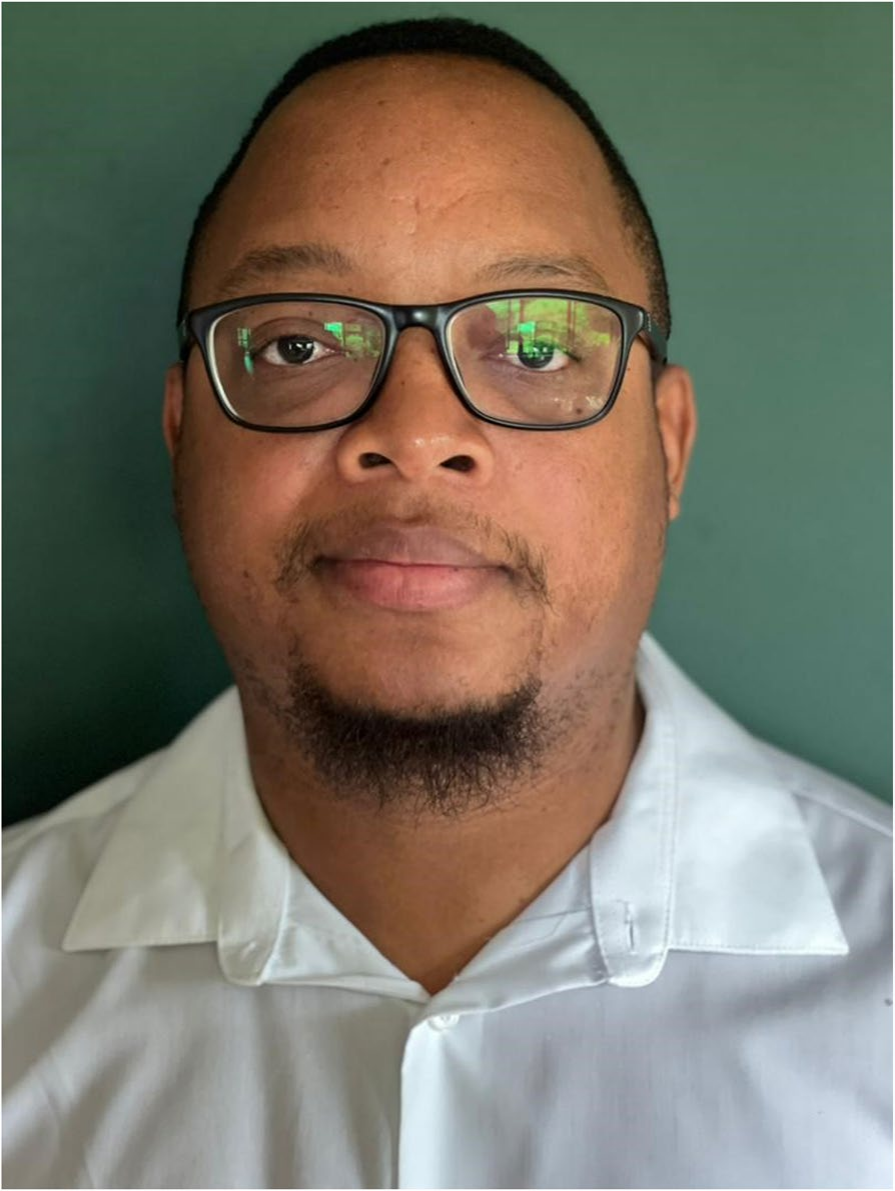
Kondwani Charles Jambo is a Group Leader at the Malawi-Liverpool-Wellcome Programme and a Senior Lecturer at the Liverpool School of Tropical Medicine. He is an immunologist, with an interest in natural and vaccine-induced immunity at the individual and population level. His research aims at addressing public health problems relevant to low-income settings. His research has provided critical insights on the extent of SARS-CoV-2 population exposure and immunity in Malawi. He has provided expert advice towards formulation and revision of the national COVID-19 vaccination policy. Dr Jambo is a recipient of the Wellcome Intermediate Fellowship and the MRC African Research Leader award

Figure 9 shows the biography of Dr. Jambo.

**Fig. 10:**
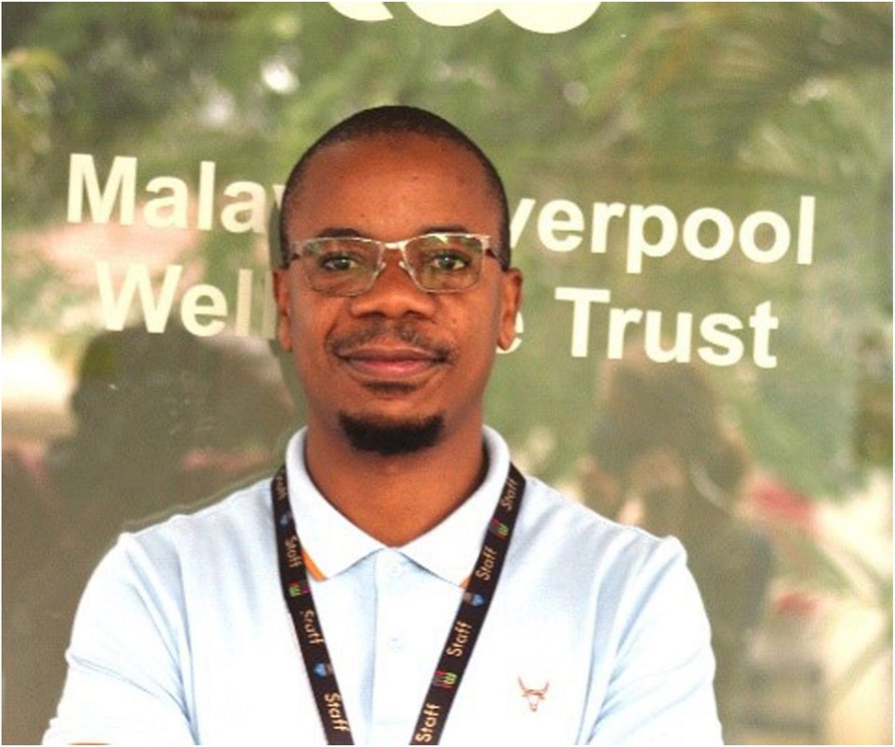
Dr Latif Ndeketa is Principal Research Associate at the Malawi-Liverpool-Wellcome Trust Clinical Research Programme (MLW). At MLW, he is the Deputy Group head of the Infectious Disease Epidemiology Group and co-leads the Vaccines Theme. Latif contributes to the accelerated reduction of morbidity and mortality of vaccine preventable diseases in low-income settings through novel surveillance methods for vaccine programs. He leads clinical research studies, provides scientific advice to relevant government and private institutions, and is an active member of several national technical advisory committees. He is a Medical Doctor with a Masters in Vaccinology and Drug Development, and a Masters in Epidemiology

Figure 10 shows the biography of Dr. Ndeketa.

#### COVID-19 vaccine availability in Africa

Vaccines were regarded from early on in the pandemic as one of the pillars in the fight against COVID-19 [150, 151]. The first vaccine to receive the World Health Organization (WHO) Emergency Use Listing (EUL) status was the Pfizer/BioNTech mRNA vaccine on 31 December 2020 [152]. To promote equitable access to vaccines for low-middle income countries (LMICs), COVAX was established as a collaboration co-led by Gavi, Coalition for Epidemic Preparedness Innovations (CEPI), and the WHO with UNICEF as the key delivery partner [153]. COVAX’s biggest beneficiaries are countries in Africa and South Americas. The next COVID-19 vaccine to be accessible was the AstraZeneca vaccine, which was granted EUL approval by the WHO on 15 February 2021 [154]. The AstraZeneca vaccine approval was a pivotal moment in efforts to accelerate vaccine access to Africa due to its affordability, easier storage, and transportation [155].

Côte d’Ivoire and Ghana were the first recipients of the first-round allocation by COVAX in Africa on the 1st of March 2021 and more countries soon followed [156]. However, as of 14 February 2022, only 26.1% of the population in Africa had received at least one dose in contrast to 61.8% for the rest of the world’s population [157]. According to WHO, over 670 million doses have been received in the 53 African states to date, with 64% of the doses from COVAX, 29% from bilateral agreements between countries, and 6% from the African Union’s African Vaccine Acquisition Trust (AVAT) [158]. The COVID-19 vaccines have included AstraZeneca (31%), Sinopharm (20%), Sinovac (18%),Pfizer/ BioNTech (13%), Janssen (11%), Moderna (5%), Sputnik (2%), and Covaxin vaccines (0.1%) [158].

#### COVID-19 vaccine coverage in Africa

Only 26% of the population in Africa has received at least one dose of COVID-19 vaccines in contrast to 62% of the global coverage of at least one dose [158, 159]. This represents a ratio of 11.8 per 100 people that are fully vaccinated with COVID-19 vaccines on the continent. Of the 677 million COVID-19 vaccines received in Africa, about 364 million doses (54%) have been administered and 163 million have completed the primary series doses [158].

In May 2021, the WHO set targets for countries to have 10% of their population vaccinated by end September 2021, 40% of their population by end December 2021, and 70% by mid-2022 [160]. By the end of September 2021, only 16 African countries had met the target to fully vaccinate 10% of their total population eligible for vaccines. Only seven countries achieved 40% vaccination coverage by end December 2021, with Seychelles, Mauritius, and Morocco able to vaccinate over 60% of their population [158, 161]. Majority of the countries (*n* = 46) missed the 40% target, including some of Africa’s largest populations such as Nigeria, Ethiopia, and Democratic republic of Congo as they managed to vaccinate only <5% of their population. Tanzania and Burundi were the last two countries to commence their COVID-19 vaccination campaigns, due to policy divergence from the WHO recommendations. Following alignment with WHO policy recommendation, the countries began COVID-19 vaccination, in July 2021 for Tanzania using the single-shot Johnson & Johnson vaccine [162] and in October 2021 for Burundi with Sinopharm vaccine [163]. However, Eritrea remains the only African country yet to start their COVID-19 vaccination campaign [158]. Judging from the current trend in vaccine uptake, it is only a few countries that are set to have 70% of their population vaccinated by mid-2022.

#### Barriers to COVID-19 vaccine uptake in Africa

The low vaccination coverage and uptake in Africa is multifactorial, but with most of the challenges stemming from inequitable distribution of vaccines [164]. Firstly, the observed low utilization rate of COVID-19 vaccines in Africa is partly due to the expected lag in time between in-country receipt of vaccines and time of vaccination, as there are preparatory and logistical steps at a national level that need to occur. Vaccine rollout is accompanied by key steps that include but not limited to training and supervision of healthcare workers, assessment of vaccine cold chain capacity, and regulatory and safety surveillance [165, 166]. It has been demonstrated that COVID-19 vaccine utilization increases as vaccines are accessible to the general public [158]. Secondly, the inconsistent supply of COVID-19 vaccines to Africa, due to lack of equitable access to vaccines, is a major barrier to vaccine uptake. The vaccine supply challenge was initially caused by agreements between high-income countries and manufacturers where doses were reserved by wealthier nations before the vaccines became available [167]. This was coupled with vaccine hoarding where wealthy countries had surplus vaccines than needed for their population while countries in Africa were still waiting for supplies [168]. Furthermore, exports to Africa of the AstraZeneca (COVISHIELD) vaccine by Serum Institute of India (SII), which had mandate to produce the vaccine for LMICs at lower cost [169], were blocked by the Indian government when the country experienced its second wave that had a high case fatality rate [170]. This led to shortage of supply of vaccines to Africa as SII is one of the main vaccine suppliers to the COVAX facility [171]. Thirdly, across the globe, reluctance of people to receive safe and recommended available vaccines, termed vaccine hesitancy, has slowed down the consumption rate of COVID-19 vaccines. Across the continent, it has been a common trend that the vaccines donated to African countries by wealthier countries have had a short shelf life, which has led to the inevitable expiry and destruction of the vaccines in the recipient country because countries are unable to adequately plan for vaccination campaigns [172]. This has fuelled distrust and perpetuated vaccine hesitancy. In addition, the risk perception of the people on the COVID-19 pandemic, considering the less severe nature of the pandemic in Africa compared to the Americas and Europe [150], has contributed to vaccine hesitancy. Lastly, country-level preparedness has impacted vaccine uptake in Africa. Vaccine arrival in some countries was met by national deployment and vaccination plans that were not updated, which is a pre-requisite for countries to apply for implementation funds [173]. Furthermore, public engagement to encourage COVID-19 vaccination has been suboptimal in reaching rural communities in some countries, this is in contrast to the robust immunization campaigns most African countries are known for, that have led to the high childhood vaccination coverage across the continent [174].

#### Potential enablers to COVID-19 vaccine uptake in Africa

Africa is the second most populous continent [175], yet it remains the only continent that lacks local technological capacity for COVID 19 vaccine manufacturing. The COVID-19 pandemic has been a lesson for African countries to urgently invest in local technological capacity for vaccine manufacturing. This could in part also help solve the problem of vaccine hesitancy, as the recipients are more likely to have trust in locally manufactured vaccines. At a global level, COVID-19 vaccine manufacturers should be incentivized to increase vaccine access through the sharing of vaccine manufacturing technology in order for the vaccines to be locally produced in Africa while maintaining their intellectual property rights similar to the recent agreement between Moderna, WHO, and Afrigen Biologics and Vaccines in Cape Town, South Africa [176]. Recently, Germany’s BioNTech announced plans to send vaccine production units in shipping containers to Africa, with the initial facility expected to arrive in the second half of 2022, and manufacturing starting about 12 months after delivery [177]. As vaccine supply to Africa drastically increased around mid-August 2021, we witnessed a similar rise in vaccine utilization in the region [158]. However, the gap between supply and uptake has exponentially increased since November 2021 [158], which underlines that Africa’s current primary challenge is both supply and uptake. With improved vaccine supply, countries must accelerate uptake through improved demand creation. There should be contextualized and country-tailored support from multilateral partners to understand the challenges faced by each country and their root causes in order to overcome human, financial, and technical assistance needs. Moreover, there is a need for joint coordination efforts between governments in Africa to find data-driven innovative methods to lower vaccine hesitancy and increase uptake, as this has potential to derail the highly successful childhood vaccination programs in Africa.

**Competing interests**

The authors declare that they have no competing interests.

**Authors’ contributions**

All authors were involved in drafting, editing, and revising the manuscript and agreed to its publication. All authors read and approved the final manuscript.

## Impact of the COVID-19 pandemic on essential health and other social services in Africa

### Rhoda K. Wanyenze and Ambrose O. Talisuna

**Fig. 11:**
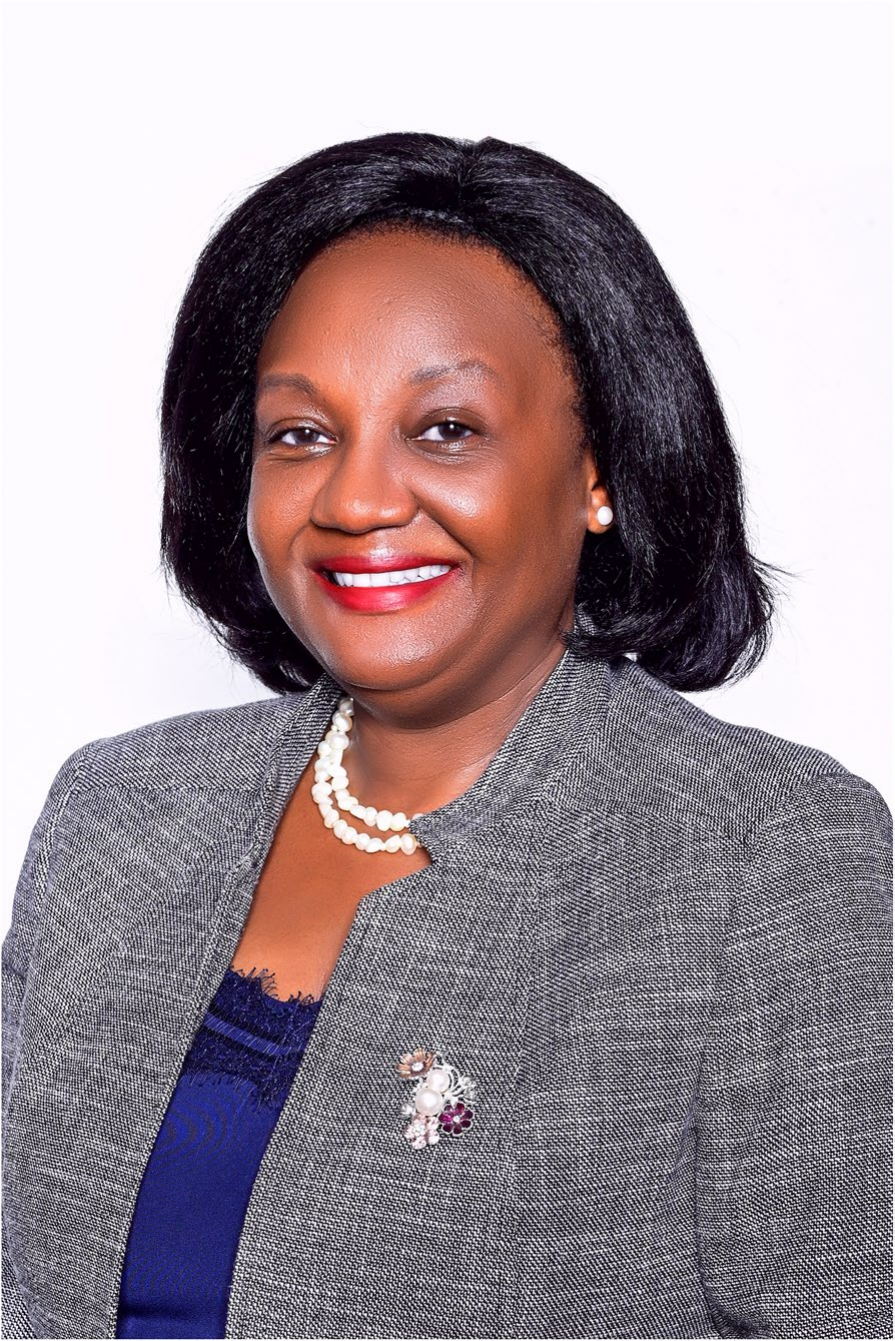
Dr. Rhoda Wanyenze, MBChB, MPH, PhD, is Dean of the School of Public Health and a Professor in the Department of Disease Control and Environmental Health at Makerere University. Dr. Wanyenze has vast experience in public health research, capacity building, and program management, especially in infectious diseases, sexual and reproductive health, and health systems. She has led a wide network of research partnerships with academic institutions and ministries of health in Africa and is Principal Investigator for a study evaluating the Covid-19 response in five countries in Africa. She has served on Boards of several organizations in Uganda and globally

Figure 11 shows the biography of Prof. Wanyenze.

**Fig. 12:**
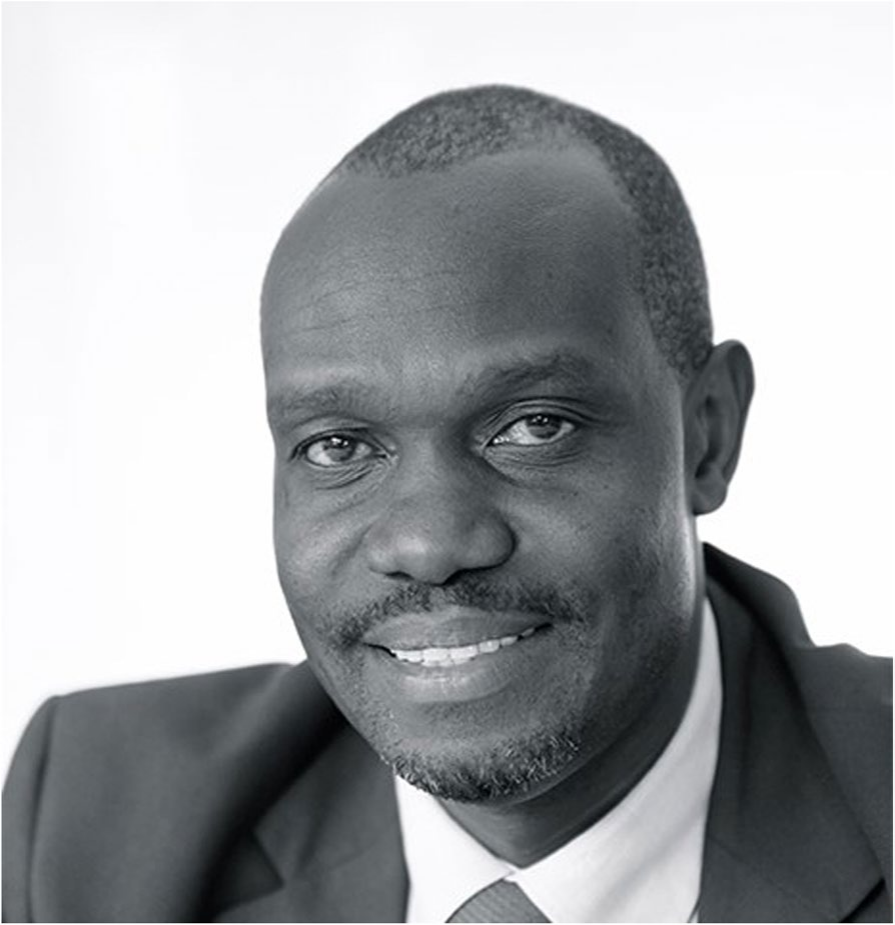
Ambrose has medical degree in human medicine, a master’s degree in epidemiology and a PhD in biomedical sciences. Between 1996 and 2011, Ambrose held senior management, leadership and scientific positions at the Uganda Ministry of Health, the Institute of Tropical Medicine, Antwerp and the Medicines for Malaria Venture (MMV). He has been field coordinator for multi-country malaria clinical trials and a senior fellow of the European and Developing Countries Clinical Trials Partnership (EDCTP). Between 2011 and early, 2016, he was regional scientific director for East Africa of the Worldwide Antimalarial Resistance Network (WWARN) and later senior clinical research fellow with the University of Oxford-KEMRI Wellcome Trust Programme. Since 2016, Ambrose has been regional advisor for health security and International Health Regulations, spearheading the efforts to build and sustain capacities to better prevent, detect and respond to infectious disease epidemics, pandemics and other public health threats before they spread

Figure 12 shows the biography of Dr. Talisuna.

#### Introduction

Sub-Saharan Africa has the highest burden of public health events, including health and humanitarian emergencies because of its bio-geographical location. Each year, the World Health Organization Africa Region confronts over 100 health emergencies [178]. Public health threats originating from the human-animal and environmental interface occur frequently [179]. Concomitantly, climate-related events, including prolonged droughts, food insecurity, destructive floods, and cyclones, are increasing [180]. The African continent is also affected by many protracted humanitarian crises. Mass refugee migration, internal population displacements, trade practices, and cross-border movements provide opportunities for spreading infectious diseases [178].

The COVID-19 pandemic has unraveled significant gaps in emergency preparedness and health systems resilience worldwide. The pandemic has infected millions of people and caused over hundred thousand deaths in Africa [198]. Early reports from low- and middle-income countries show interruption of critical disease services for HIV, TB, and malaria as well as maternal and child health programs [181, 182]. Additionally, the public health and social measures (PHSMs) to contain the pandemic have significantly interrupted trade and supply chains and hospitality and recreational industries [183]. Consequently, stock markets have plunged, unemployment rates have risen, and economic gains have been reversed [184, 185]. This is a stark reminder about the urgent need to build resilient health systems and strengthen the International Health Regulations (IHR 2005) capacities.

#### Effects of COVID-19 on health service delivery, utilization, and outcomes

Research on the unintended consequences of Covid-19 on the African continent is still limited; however, emerging evidence shows devastating effects of the COVID-19 pandemic on economies and major disruptions to essential health services, livelihoods, and education, among others [186–188]. The COVID-19 pandemic has clearly demonstrated how public health emergencies can affect all four interconnected health system performance attributes. COVID-19 has affected access capacity—the ability to overcome barriers (physical, financial, and socio-cultural). It has also affected quality capacity—the ability to provide services as expected. Additionally, it has affected demand capacity—the ability to provide services expected by populations [189]. Importantly, the pandemic has brought to the surface inadequacies in resilience capacity—the ability to sustain the provision of essential health services during shock events. The resilience of a health system is driven by the need to ensure continuity of essential service provision. Resilience brings together the work of emergency preparedness and health systems-health security and universal health coverage (UHC) goals [53, 190].

The fear of COVID-19 affected health service delivery and utilization because health workers and communities avoided going to health facilities [191]. The repurposing and diversion of human, financial, and material resources to the COVID19 response deprived other priority health programs. However, the biggest effects have perhaps been due to the negative consequences of the PHSMs adopted to prevent COVID-19, and the challenges with prioritization of investments in health. Importantly, the PHSMs to contain the pandemic such as the nationwide lockdowns and other movement restrictions, closure of schools, discontinuation of community health service outreaches for immunization, family planning, and other health services, as well as closure of formal and informal services such as trade have created devastating effects on health and other services [192].

Restriction of movement of health service providers led to complete absenteeism or late arrival to duty station. Inadequate infection prevention control (IPC) resulted in COVID-19 infections among health workers and further exacerbated the absenteeism [193]. The disruption of health services was further exacerbated by the fragile health systems and the absence of clear plans and guidelines for the continuity of essential health services [194]. For example, the poor investment in human resource surge capacity led to repurposing of health workers to conduct surveillance, contact tracing, and other activities as part of the COVID-19 response and affected the delivery of the other health services. Space and infrastructure for some health services was repurposed too. In Uganda, for example, mental health wards were used as isolation centers for COVID-19 at the onset of the pandemic while in Cameroon tertiary health facilities where designated to exclusively care for COVID-19 cases, yet these facilities offer other essential health services [182, 194]. Laboratory equipment and personnel across various countries such as the GeneXpert were repurposed to Covid-19 testing [195].

Movement and travel restrictions also affected the delivery of health commodities and supplies across various levels of service delivery, with resultant stockout of essential supplies [194, 195]. The disruption of health services had immediate effects on service utilization, leading to negative outcomes such as increased mortality among women during childbirth, poor coverage of childhood immunization services, loss to follow-up among patients in chronic care clinics such as HIV, tuberculosis, and non-communicable disease clinics, including diabetes and hypertension clinics [194, 196].

Although countries developed policies and guidelines for the continuity of essential health services [196, 197], including data analysis to track and address disruptions, the pace of adoption of these interventions was quite varied, with significant delays, varied levels of disruptions, and outcomes across many countries [197]. To counter the disruptions, health workers were trained, and new service delivery models were adopted or expanded, for example, multi-month drug refills for chronic care patients, home-based delivery of supplies using motorcycles, telephone-based counseling, etc. These interventions led to recovery of the services where they were applied while some services that did not receive much attention lagged [194, 197, 198].

The International Planned Parenthood Federation (IPPF) report of 2021 revealed declines in service delivery evidenced by closure of mobile clinic services globally, with the African region experiencing the largest number of clinic closures (447 closures) [199]. There were reports of vaccination clinics stopped in Zimbabwe and a resurgence of measles in DRC [200]. In Ethiopia, a national level study of effects on reproductive, maternal, newborn, child, and adolescent health and older people (RMNCAAH) services showed minimal disruptions of the RMNCAAH indicators [198]. However, a study in Northern Ethiopia revealed increased institutional caesarian sections and still births and in the number of under-5 children with malnutrition as well as a decrease in HIV testing and enrolment on to antiretroviral therapy among HIV-infected individuals [201]. These national and subnational differences reflect the varied disruptions across regions and different services, and the need for disaggregated analyses within countries for targeted mitigation measures where needed [194]. In Kwazulu Natal, South Africa, there was a 36% decline in clinic attendance and 50% decline in hospital admissions of children in April–June 2020, although the changes were less for the hospital births and testing of HIV-exposed infants. Although short-lived, there was a 47% increase in neonatal deaths in May 2020 [202]. In Rwanda, there was a reduction in malaria testing in health facilities among patients by 4.32 per 1000 population while testing in the community increased by 2.38 per 1000 population monthly, due to challenges and delays in accessing care at facilities [203]. The findings in Kenya were mixed, with declines in some services while utilization of other services increased or did not show significant changes. While bed occupancy reduced by 25%, measles vaccination increased by 44% [204]. In another study, the majority of women in Kenya and Burkina Faso did not change their contraceptive status and were in fact more likely to adopt a method if the status changed (25 and 13%, respectively). Discontinuation was lower at 6 and 5%, respectively [205]. In Cameroon and DRC, several RMNCAAH in 2020 were comparable to 2019 and some where even higher, perhaps due to the service adaptations implemented with a focus on sustaining these services [198].

The disruptions to health and service access were more pronounced among the most vulnerable populations. For example, the absence of a functional referral and ambulance system, yet the poorest communities and families could not afford car hire to access emergency health services [206]. Similarly, with migration to virtual approaches and e-learning, the poorest and most disadvantaged families could not have access to these services because they neither have electronic devices nor internet access to support learners [207]. Limited safety nets for the most vulnerable has further aggravated the inequities within and across countries [190]. Worryingly, despite the major impact of COVID 19 on mental and social welfare, mental health and psychosocial services were not prioritized. Consequently, negative mental health effects including depression, anxiety, post-traumatic stress, and behavior trouble have increased due to the impact of movement restrictions and socio-economic challenges [21, 208–212].

On the other hand, there were investments to improve critical care capacity, laboratory infrastructure (diagnostics, genomic surveillance, pooled scientific, and other resources, e.g., training in PCR for COVID-19 testing), emergence of pooled procurement and distribution networks, and renewed commitments towards local production and public-private partnerships across the continent [213, 214], which if sustained could lead to longer-term improvements in systems and care across various disease conditions. The longer-term effects of these health service disruptions and investments on the achievement of the sustainable development goals (SDGs) and UHC targets are yet to be fully assessed and are an important area for further research. Additionally, the disruptions of other critical determinants of health including education, livelihood, and increased poverty among the most vulnerable groups may have longer-lasting effects on health and health outcomes and should be further investigated.

#### Increased gender-based and domestic violence

An Oxfam report shows an undeniable increase in gender-based violence (GBV) during the COVID-19 pandemic around the world to which too many governments and donors are not doing enough to tackle. The Oxfam report, titled “The Ignored Pandemic: The Dual Crisis of Gender-Based Violence and COVID-19”, showed the number of calls made by survivors to domestic violence hotlines in ten countries during the first months of lockdown. The data reveals a 25–111 percentage surge, in Tunisia (43%), Somalia (50%), and South Africa (69%) [215]. In many households, coronavirus created a “perfect storm” of social and personal anxiety, stress, economic pressure, social isolation, including with abusive family members or partners, and rising alcohol and substance use, resulting in increases in domestic abuse [216]. Worryingly, not enough countries have acted with sufficient seriousness to tackle the GBV pandemic. Even before the surge in GBV cases sparked by the pandemic, in 2018 alone, over 245 million women and girls were subjected to sexual or physical violence by an intimate partner—a greater number than the global total of coronavirus cases (199m) between October 2020 and October 2021 [215].

#### Disruption of education

School closures were instituted across many countries in Africa in March–June 2020, and most of these remained closed for prolonged periods with the longest closure of 22 months and a predicted learning deficit of 2.8 years in Uganda [217]. Burundi on the other hand did not close schools due to Covid-19 while Tanzania closed schools for a few months [218]. In an assessment of school reopening in 40 Global Partnership for Education (GPE) countries, >60% opened after >200 days of closure [218]. While there was increased adoption of e-learning across countries, this mostly favored high-income households in urban areas—those that had access to internet and e-learning tools. Children got involved in creative household work and trade, which may enhance learning but also has the unintended consequences in terms of child labor and exploitation, with some learners dropping out of school due to the attraction to petty trade [218].

Further, there have been widespread reports of early marriages and pregnancies among the girl child, with many young mothers failing to return to school or struggling to cope with the demands of school while caring for their children [217–220]. Overall, learning was disrupted for most of the learners with a lack of progression to the next level, with reports of anxiety and other mental health issues driven by stress and increased use of alcohol and drugs [217]. There have also been reports of teachers losing jobs due to downsizing or school closures while others opted for more lucrative forms of employment [217–220].

#### Conclusion

Although Africa reported the least number of cases and deaths of all the continents, it is our considered view that the COVID-19 pandemic and its related PHSMs have had mixed outcomes in Africa. Major disruptions occurred in the delivery of essential health and other social services, and negative socioeconomic consequences that could reverse decades of economic progress in Africa. Worryingly, the discontinuation of education will have major consequences that need to be tracked and addressed in the medium to long term. Moreover, there have been major effects on household and individual incomes and increasing unemployment. Effects on mental health and gender-based violence will need African countries to carefully build a cadre of mental health experts and increase the number of facilities offering mental health care and addressing GBV.

Finally, some of the effects could be sustained for a long time and potentially affect the attainment of the sustainable development goals and UHC targets if remedial actions are not timely. The lessons from the COVID-19 response should inform future epidemic and pandemic preparedness and response strategies and plans—specifically ensuring resilience of health and other systems to mitigate the negative effects of health emergencies. Continuity of essential health services and mitigation of unintended negative consequences during shock events should be fully integrated into the policies and strategies for health emergency preparedness and response.

**Competing interests**

Both authors declare that they have no competing interests.

**Authors’ contributions**

AOT has no conflict of interest. RW and AOT conceptualized the idea, and RW wrote the first draft of the manuscript that was reviewed by AOT. Both authors have reviewed and approved the final manuscript.

## References

[1] Huang C, Wang Y, Li X, Ren L, Zhao J, Hu Y, et al. Clinical features of patients infected with 2019 novel coronavirus in Wuhan, China. Lancet. 2020;395(10223):497–506. [cited 2022 Feb 26]. Available from: https://pubmed.ncbi.nlm.nih.gov/31986264/. 10.1016/S0140-6736(20)30183-5 PMC7159299 31986264

[2] Allam Z. The first 50 days of COVID-19: a detailed chronological timeline and extensive review of literature documenting the pandemic. In: Surveying the Covid-19 Pandemic and its Implications. Elsevier; 2020. p. 1–7. [cited 2022 Feb 26]. Available from: https://www.ncbi.nlm.nih.gov/pmc/articles/PMC7378494/.

[3] MPHonline. Outbreak: 10 of the Worst Pandemics in History. Masters of Public Health Online. 2020. 1–7. [cited 2022 Feb 26]. Available from: https://www.mphonline.org/worst-pandemics-in-history/.

[4] Pak A, Adegboye OA, Adekunle AI, Rahman KM, McBryde ES, Eisen DP (2020). Economic consequences of the COVID-19 outbreak: the need for epidemic preparedness. Front Public Heal..

[5] Robert Wood Johnson Foundation. The impact of coronavirus on households across America. 2020 [cited 2022 Feb 20]. Available from: https://www.rwjf.org/en/library/research/2020/09/the-impact-of-coronavirus-on-households-across-america.html.

[6] United Nations. Human Development Index (HDI) | Human Development Reports. United Nations Development Programme. 2018 [cited 2022 Feb 20]. Available from: https://hdr.undp.org/en/content/human-development-index-hdi.

[7] Pearson CAB, van Schalkwyk C, Foss AM, O’Reilly KM, Pulliam JRC. Projected early spread of COVID-19 in Africa through 1 June 2020. Eurosurveillance. 2020;25(18). [cited 2022 Feb 26]. Available from: https://www.ncbi.nlm.nih.gov/pmc/articles/PMC7219031/. 10.2807/1560-7917.ES.2020.25.18.2000543 PMC7219031 32400361

[8] World Bank. Tackling Vaccine Inequity for Africa. 2021 [cited 2022 Feb 26]. Available from: https://blogs.worldbank.org/voices/tackling-vaccine-inequity-africa.

[9] Review WP. Population by Continent 2021. 2021 [cited 2022 Feb 26]. Available from: https://worldpopulationreview.com/continents.

[10] United Nations. Africa needs to ramp up COVID-19 vaccination rate six-fold | | UN News. [cited 2022 Feb 27]. Available from: https://news.un.org/en/story/2022/02/1111202.

[11] World Health Organization. WHO coronavirus (COVID-19) Dashboard. WHO Coronavirus (COVID-19) dashboard with vaccination data. WHO. 2021. 1–5. [cited 2021 Dec 29]. Available from: https://covid19.who.int/info.

[12] Adams J, MacKenzie MJ, Amegah AK, Ezeh A, Gadanya MA, Omigbodun A, et al. The conundrum of low covid-19 mortality burden in Sub-Saharan Africa: myth or reality?. Vol. 9, Global Health Science and Practice. Global Health: Science and Practice; 2021; 433–43. [cited 2022 Mar 2]. Available from: https://www.ghspjournal.org/content/9/3/433. 10.9745/GHSP-D-21-00172 PMC8514030 34593571

[13] United Nations. How many countries in Africa? - Worldometer. WorldOMeter. 2021 [cited 2022 Feb 21]. Available from: https://www.worldometers.info/geography/how-many-countries-in-africa/.

[14] The Fund for Peace. Fragile States Index Annual Report 2020. Fund Peace. 2021;4–5. [cited 2022 Feb 20]. Available from: www.fundforpeace.org.

[15] World Bank. Classification of fragile and conflict-affected situations. World Bank. 2020 [cited 2022 Feb 20]. Available from: https://www.worldbank.org/en/topic/fragilityconflictviolence/brief/harmonized-list-of-fragile-situations.

[16] Ndwandwe D, Wiysonge CS. COVID-19 vaccines. Curr Opin Immunol. 2021; 71:111–6. [cited 2022 Feb 20]. Available from: https://www.who.int/emergencies/diseases/novel-coronavirus-2019/covid-19-vaccines. 10.1016/j.coi.2021.07.003 PMC8272971 34330017

[17] World Health Organization. WHO SAGE roadmap for prioritizing uses of Covid 19 vaccines in the context of limited supply. Who. 2020;(September):1–13. [cited 2022 Feb 20]. Available from: https://www.who.int/news/item/21-01-2022-updated-who-sage-roadmap-for-prioritizing-uses-of-covid-19-vaccines.

[18] World Health Organization. Evidence to recommendations for COVID-19 vaccines: evidence framework. 2020;1–9. [cited 2022 Feb 20] Available from: https://www.who.int/publications/i/item/WHO-2019-nCoV-SAGE-Framework-Evidence-2020-1.

[19] Feinmann J. Covid-19: Global vaccine production is a mess and shortages are down to more than just hoarding. BMJ. 2021;375. [cited 2022 Feb 20] Available from: https://www.bmj.com/content/375/bmj.n2375. 10.1136/bmj.n2375 34711605

[20] Shang Y, Li H, Zhang R (2021). Effects of pandemic outbreak on economies: evidence from business history context. Front Public Heal..

[21] OECD policy responses to coronavirus (COVID-19). COVID-19 and Africa: socio-economic implications and policy responses. 2020. https://www.oecd.org/coronavirus/policy-responses/covid-19-and-africa-socio-economic-implications-and-policy-responses-96e1b282/.

[22] WHO. COVID-19 Public Health Emergency of International Concern (PHEIC) Global research and innovation forum. 2020 12th February 2020; Available from: https://www.who.int/publications/m/item/covid-19-public-health-emergency-of-international-concern-(pheic)-global-research-and-innovation-forum.

[23] WHO. Strategic Response to COVID-19 in the WHO African Region | WHO | Regional Office for Africa. Strategic Response to COVID-19 in the WHO African Region. 2021;3. https://www.afro.who.int/publications/strategic-response-covid-19-who-african-region. Accessed 6 Nov 2021.

[24] Springer Nature. Top 20 infectious diseases | Nature Index 2021 infectious disease | Supplements | Nature Index. https://www.natureindex.com/supplements/nature-index-2021-infectious-disease/tables/diseases. Accessed 9 Mar 2022.

[25] Sarfraz M, Ozturk I, Shah SGM (2022). Editorial: coronavirus disease (COVID-19): the impact on psychology of sustainability, sustainable development, and global economy. Front Psychol..

[26] Ibn-Mohammed T, Mustapha KB, Godsell J, Adamu Z, Babatunde KA, Akintade DD (2021). A critical review of the impacts of COVID-19 on the global economy and ecosystems and opportunities for circular economy strategies. Resour Conserv Recycl..

[27] Organisation for Economic Cooperation and Development (OECD). The impact of the coronavirus (COVID-19) crisis on development finance. Tackling coronavirus Contrib to a Glob effort. 2020;100:468–70. https://www.oecd.org/coronavirus/policy-responses/the-impact-of-the-coronavirus-covid-19-crisis-on-development-finance-9de00b3b/. Accessed 9 Mar 2022.

[28] Udoakang A, Oboh M, Henry-Ajala A, Anyigba C, Omoleke S, Amambua-Ngwa A, et al. Low COVID-19 impact in Africa: the multifactorial Nexus. AAS Open Res. 2021;4:47. doi:10.12688/aasopenres.13261.1.

[29] UNDP Regional Bureau for Africa. Graduation of African Least Developed Countries ( LDCs ) : emerging issues in a new development landscape. 2021. https://www.africa.undp.org/content/rba/en/home/library/reports/graduation-of-african-least-developed-countries%2D%2Dldcs%2D%2D%2D%2Demergin.html. Accessed 16 Nov 2021.

[30] Gilbert M, Pullano G, Pinotti F, Valdano E, Poletto C, Boëlle PY (2020). Preparedness and vulnerability of African countries against importations of COVID-19: a modelling study. Lancet..

[31] World Health Organization. WHO coronavirus (COVID-19) dashboard. WHO coronavirus (COVID-19) dashboard with vaccination data. WHO. 2021;:1–5. https://covid19.who.int/info. Accessed 29 Dec 2021.

[32] Ogden N, AbdelMalik P, Pulliam J. Emerging infectious diseases: prediction and detection. Canada Commun Dis Rep. 2017;43:206–11. doi:10.14745/ccdr.v43i10a03. 10.14745/ccdr.v43i10a03 PMC5764723 29770047

[33] Sengupta M, Roy A, Ganguly A, Baishya K, Chakrabarti S, Mukhopadhyay I (2021). Challenges encountered by healthcare providers in COVID-19 times: an exploratory study. J Health Manag..

[34] Perez Perez GI, Talebi Bezmin Abadi A. Ongoing challenges faced in the global control of COVID-19 pandemic. Arch Med Res. 2020;51:574–6. doi:10.1016/j.arcmed.2020.04.016. 10.1016/j.arcmed.2020.04.016 PMC7188630 32446538

[35] Pius T, Nabaasa S, Kusiima N, Eze ED, Yashim BJ, Robinson S (2020). Combating the spread of COVID-19, the challenges faced and way forward for the international community: a review. OALib..

[36] Allam Z. The first 50 days of COVID-19: a detailed chronological timeline and extensive review of literature documenting the pandemic. In: Surveying the Covid-19 Pandemic and its Implications. Elsevier; 2020. p. 1–7. 10.1016/b978-0-12-824313-8.00001-2.

[37] WHO. WHO coronavirus (COVID-19) dashboard. WHO coronavirus (COVID-19) dashboard with vaccination data. Who. 2021;:1–5. https://covid19.who.int/?gclid=Cj0KCQiAhMOMBhDhARIsAPVml-GMXn-jpn3ENocYrWkGtzD6UCl1ToM6ZmbCiGCskzuqL3JkAEwZbqgaAs35EALw_wcB. Accessed 15 Nov 2021.

[38] World Health Organization. Tracking SARS-CoV-2 variants. Who. 2021;:https://www.who.int/en/activities/tracking-SARS-Co. https://www.who.int/en/activities/tracking-SARS-CoV-2-variants/. Accessed 10 Jan 2022.

[39] Statista. Africa: COVID-19 Delta variant cases 2021 | Statista. 2021. https://www.statista.com/statistics/1249798/number-of-sars-cov-2-delta-variant-cases-in-africa-by-country/. Accessed 10 Jan 2022.

[40] OMS. Enhancing Readiness for Omicron (B.1.1.529): Technical Brief and Priority Actions for Member States. 2021. https://www.who.int/publications/m/item/enhancing-readiness-for-omicron-(b.1.1.529)-technical-brief-and-priority-actions-for-member-states. Accessed 10 Jan 2022.

[41] World Health Organization. Classification of Omicron (B.1.1.529): SARS-CoV-2 Variant of Concern. WHO. 2021;337. https://www.who.int/news/item/26-11-2021-classification-of-omicron-(b.1.1.529)-sars-cov-2-variant-of-concern. Accessed 10 Jan 2022.

[42] Zoa-Assoumou S, Ndeboko B, Manouana GP, Myrabelle R, Houechenou A, Bikangui R (2021). SARS-CoV-2 emerging variants in Africa: view from Gabon. The Lancet Microbe..

[43] worldometer. Population of Africa (2021) - Worldometer. Worldometer.Com. 2021. https://www.worldometers.info/world-population/africa-population/. Accessed 29 Dec 2021.

[44] Diop BZ, Ngom M, Pougué Biyong C, Pougué Biyong JN (2020). The relatively young and rural population may limit the spread and severity of COVID-19 in Africa: a modelling study. BMJ Glob Heal..

[45] International Development Association. Urban population (% of total population) - Sub-Saharan Africa | Data. World Bank Group. 2021. https://data.worldbank.org/indicator/SP.URB.TOTL.IN.ZS?locations=ZG. Accessed 29 Dec 2021.

[46] Gilbert M, Pullano G, Pinotti F, Valdano E, Poletto C, Boëlle PY (2020). Preparedness and vulnerability of African countries against importations of COVID-19: a modelling study. Lancet..

[47] World Data. The 96 most popular travel countries. 2020. https://www.worlddata.info/tourism.php. Accessed 13 Mar 2022.

[48] Haider N, Osman AY, Gadzekpo A, Akipede GO, Asogun D, Ansumana R (2020). Lockdown measures in response to COVID-19 in nine sub-Saharan African countries. BMJ Global Health..

[49] UNICEF. School at last | UNICEF Uganda. https://www.unicef.org/uganda/stories/school-last. Accessed 12 Mar 2022.

[50] Chitungo I, Dzobo M, Hlongwa M, Dzinamarira T (2020). COVID-19: unpacking the low number of cases in Africa. Public Heal Pract..

[51] Mulu A, Bekele A, Abdissa A, Balcha TT, Habtamu M, Mihret A, et al. The challenges of COVID-19 testing in Africa: the Ethiopian experience. Pan Afr Med J. 2021;38:1–4. doi:10.11604/pamj.2021.38.6.26902. 10.11604/pamj.2021.38.6.26902 PMC7825374 33520075

[52] United Nations. Human Development Index (HDI) | Human Development Reports. United Nations Development Programme. 2018. https://hdr.undp.org/en/content/human-development-index-hdi. Accessed 20 Feb 2022.

[53] Madhi SA, Nel J (2021). Epidemiology of severe COVID-19 from South Africa. In The Lancet HIV. Elsevier..

[54] Adams J, MacKenzie MJ, Amegah AK, Ezeh A, Gadanya MA, Omigbodun A, et al. The conundrum of low covid-19 mortality burden in Sub-Saharan Africa: myth or reality? Global Health Sci Pract. 2021;9:433–443. doi:10.9745/GHSP-D-21-00172. 10.9745/GHSP-D-21-00172 PMC8514030 34593571

[55] World Data. Average age by country. World Data. 2021. https://www.worlddata.info/average-age.php. Accessed 7 Mar 2022.

[56] The World Bank. Net official development assistance and official aid received (current US$) - Ethiopia. The World Bank. 2019. https://data.worldbank.org/indicator/DT.ODA.ALLD.CD?locations=T6. Accessed 12 Mar 2022.

[57] The World Bank. $75 million grant to support reforms and mitigate pandemic impact in Sierra Leone. Press Release. 2021. https://www.worldbank.org/en/news/press-release/2021/12/16/75-million-grant-to-support-reforms-and-mitigate-pandemic-impact-in-sierra-leone. Accessed 12 Mar 2022.

[58] Jia S, Williamson CR (2019). AID, policies, and growth: why so much confusion?. Contemp Econ Policy..

[59] Melesse MT. International aid to Africa needs an overhaul. Tips on what needs to change. 2021. https://reliefweb.int/report/world/international-aid-africa-needs-overhaul-tips-what-needs-change. Accessed 12 Mar 2022.

[60] Park J-D. Assessing the role of foreign aid, donors and recipients. In: Re-Inventing Africa’s Development. Springer International Publishing; 2019. p. 37–60.

[61] Njoroge PN. The impact of foreign aid on development in Africa: a comparative study of Ghana and Angola. 2020. http://erepository.uonbi.ac.ke/handle/11295/154427. Accessed 12 Mar 2022.

[62] WHO. WHO declares Public Health Emergency on novel coronavirus. 2020 30th January 2020 31st January 2022]; Available from: https://www.paho.org/en/news/30-1-2020-who-declares-public-health-emergency-novel-coronavirus.

[63] WHO. COVID-19 strategy update. 2020 14th April 2020 31st January 2022]; Available from: https://www.who.int/docs/default-source/coronaviruse/covid-strategy-update-14april2020.pdf.

[64] Xylogiannopoulos KF, Karampelas P, Alhajj R (2021). COVID-19 pandemic spread against countries’ non-pharmaceutical interventions responses: a data-mining driven comparative study. BMC Public Health.

[65] World Health Organization, W., Advice for the public: coronavirus disease (COVID-19), in Corona Virus Disease. 2021: Geneva.

[66] Nachega JB (2021). Contact tracing and the COVID-19 response in Africa: best practices, key challenges, and lessons learned from Nigeria, Rwanda, South Africa, and Uganda. Am J Trop Med Hygiene.

[67] Ministry of Health, M., CORONA VIRUS DISEASE - 2019 (COVID-19) Preparedness and Response Plan. 2020, Ministry of Health: Uganda. p. 78.

[68] Ghana, M.o.H., Ghana COVID-19 emergency preparedness and response project & additional financing E.A.S.M.F. (ESMF), Editor. 2020: Ghana. p. 163.

[69] Kenya., M.o.H., National 2019 novel coronavirus contingency (readiness and early response) plan. 2020: Kenya. p. 34.

[70] Malawi, G.o., National COVID-19 preparedness and response plan, M.o.D.M.A.a.P.E. The Republic of Malawi, Ministry of Health, Editor. 2020: Malawi. p. 84.

[71] Ferretti, L., et al., Quantifying SARS-CoV-2 transmission suggests epidemic control with digital contact tracing. Science, 2020. 368(6491): eabb6936. 10.1126/science.abb6936 PMC7164555 32234805

[72] Firth JA (2020). Using a real-world network to model localized COVID-19 control strategies. Nature Medicine.

[73] WHO, Progress Report for the Implementation of the COVID 19 Response Plan WHO Tanzania Country Office, January to June, 2020, W.H. Organization, Editor. 2020. p. 52.

[74] Kelley, G. Overcoming Denialism from the Top: Tanzania’s COVID-19 Response. 2021 17th June 2021 31st January 2022].

[75] Uganda, D.o.F.A. General COVID-19 Travel Advisory in Operation. 2021 16/ November/ 2021 [cited 2021 19/ November/ 2021]; Available from: https://www.dfa.ie/travel/travel-advice/a-z-list-of-countries/uganda/.

[76] Nigeria, D.o.F.A. General COVID-19 Travel Advisory in Operation. 2021 20th/ October/ 2021 [cited 2021 19/ November/ 2021]; Available from: https://www.dfa.ie/travel/travel-advice/a-z-list-of-countries/nigeria/.

[77] Kenya., D.o.F.A. General COVID-19 Travel Advisory in Operation. 2021 22/ October/ 2021 [cited 2021 19/ November/ 2021]; Available from: https://www.dfa.ie/travel/travel-advice/a-z-list-of-countries/kenya/.

[78] Isaac, K., Using evidence and analysis for an adaptive health system response to COVID-19 in Uganda in 2020, in EQUINET Case study paper. 2020: Uganda. p. 26.

[79] Ministry of Health, S.A. Health Department launches COVID service portal. COVID-19 Online Resource and News Portal 2020 [cited 2020 17th July 2020]; Available from: https://sacoronavirus.co.za/2020/07/17/health-department-launches-covid-service-portal/.

[80] UIA. Covid-19: Investors donate generously towards fight against pandemic in Uganda. 2020 22/ November/ 2021]; Available from: https://www.ugandainvest.go.ug/covid-19-investors-donate-generously-towards-fight-against-pandemic-in-uganda/.

[81] James, A. African Development Bank supports continental strategy on COVID-19 with US$27.33 million. 2020 22/ November/ 2021]; Available from: https://africacdc.org/news-item/african-development-bank-supports-continental-strategy-on-covid-19-with-us27-33-million/.

[82] Eliana, D. UN provides vital supplies for Nigeria Government’s COVID-19 response. 2020 16th April 2020]; Available from: https://www.unicef.org/nigeria/press-releases/un-provides-vital-supplies-nigeria-governments-covid-19-response.

[83] Fund., T.G. Uganda’s Remarkable Response to COVID-19. 2021 The Global Fund to Fight AIDS, Tuberculosis and Malaria 2021; Available from: https://www.theglobalfund.org/en/blog/2021-03-23-ugandas-remarkable-response-to-covid-19/.

[84] UNICEF. £19 million of UK funding to Ethiopia as part of global leadership in fight against Covid-19. COVID-19 response 2020 [cited 2021 08 April 2020]; Available from: https://www.unicef.org/ethiopia/press-releases/19-million-uk-funding-ethiopia-part-global-leadership-fight-against-covid-19.

[85] Bank, T.W. Acting Early, Fast and Together: Mobilizing Efforts to Prepare and Respond to the COVID-19 Pandemic in Ethiopia. 2021 [cited 22/ November/ 2021; Available from: https://www.worldbank.org/en/results/2021/01/14/acting-early-fast-and-together-mobilizing-efforts-to-prepare-and-respond-to-the-covid-19-pandemic-in-ethiopia.

[86] Paul, S. Overcoming Covid-19 in South Africa. News-COVID-19. 2020. Available from: https://www.directrelief.org/2020/08/overcoming-covid-19-in-south-africa/.

[87] Rex M (2021). COVID-19 - WHO boosts Nigeria’s response to COVID-19, donates 26 ventilators and 3560 pulse oximeters.

[88] Africa, T.C. What Uganda has got wrong – and right – in its struggle to contain COVID-19. 2021, 2022. Available from: https://theconversation.com/what-uganda-has-got-wrong-and-right-in-its-struggle-to-contain-covid-19-163826.

[89] Rasna, W. Corruption is undermining Kenya’s COVID-19 response. 2021. Available from: https://www.one.org/africa/blog/corruption-undermining-kenya-covid19-response/.

[90] Reliefweb. Kenya: Pandemic health workers lack protection. 2021 [31st January 2022]; Available from: https://reliefweb.int/report/kenya/kenya-pandemic-health-workers-lack-protection.

[91] web, P. Lockdown didn’t work in South Africa: Why it shouldn’t happen again. 2020 [31st January 2022]; Available from: https://www.preventionweb.net/news/lockdown-didnt-work-south-africa-why-it-shouldnt-happen-again.

[92] OCHA South Sudan. South Sudan: Humanitarian Snapshot (November 2021) - South Sudan. ReliefWeb. 2021 [cited 2021 Dec 11]. Available from: https://reliefweb.int/report/south-sudan/south-sudan-humanitarian-snapshot-november-2021.

[93] OCHA South Sudan. South Sudan: Flooding Snapshot (As of 4 November 2021) - South Sudan. ReliefWeb. 2021 [cited 2021 Dec 11]. Available from: https://reliefweb.int/report/south-sudan/south-sudan-flooding-snapshot-4-november-2021.

[94] Jones FK, Wamala JF, Rumunu J, Mawien PN, Tut KM, Wohl S, et al. Successive epidemic waves of cholera in South Sudan, 2014 - 2017. medRxiv. 2020;2020.10.09.20209262. Available from: http://medrxiv.org/content/early/2020/10/13/2020.10.09.20209262.abstract. 10.1016/S2542-5196(20)30255-2 PMC7750463 33278375

[95] Azman AS, Bouhenia M, Iyer AS, Rumunu J, Laku RL, Wamala JF, et al. High hepatitis E seroprevalence among displaced persons in South Sudan. Am J Trop Med Hyg. 2017;96(6):1296–301. [cited 2017 Jul 2]. Available from: http://www.ncbi.nlm.nih.gov/pmc/articles/PMC5462562/. 10.4269/ajtmh.16-0620 PMC5462562 28719276

[96] WHO. South Sudan weekly disease surveillance bulletin 2019 | WHO | Regional Office for Africa. 2019 [cited 2019 Jun 12]. Available from: https://www.afro.who.int/publications/south-sudan-weekly-disease-surveillance-bulletin-2019.

[97] World Health Organization, United Nations Children’s Fund (UNICEF). Progress on household drinking water, sanitation and hygiene 2000-2020: five years into the SDGs. Geneva: World Health Organization; 2021. Available from: https://apps.who.int/iris/handle/10665/345081.

[98] Medecins Sans Frontiers. Hepatitis E on the rise among poor sanitary conditions in Bentiu camp - South Sudan. ReliefWeb. 2021 [cited 2021 Dec 11]. Available from: https://reliefweb.int/report/south-sudan/hepatitis-e-rise-among-poor-sanitary-conditions-bentiu-camp.

[99] Nordling, L. HIV and TB increase death risk from COVID-19, study finds—but not by much. Science. 2020;382(25):2411. Available from: https://www.sciencemag.org/news/2020/06/hiv-and-tb-increase-death-risk-covid-19-study-finds-not-much.

[100] Olu OO, Lako R, Wamala JF, Ramadan PO, Ryan C, Udenweze I, et al. What did we learn from preparing for cross-border transmission of Ebola virus disease into a complex humanitarian setting – The Republic of South Sudan? Infect Dis Poverty. 2020;9(1):40. Available from: 10.1186/s40249-020-00657-8. 10.1186/s40249-020-00657-8 PMC7170723 32312320

[101] South Sudan Ministry of Health. South Sudan - COVID-19 Intra-Action Review. WHO | Regional Office for Africa. 2020 [cited 2021 Jul 24]. Available from: https://www.afro.who.int/publications/south-sudan-covid-19-intra-action-review.

[102] WHO. New COVID-19 rapid tests a game changer for Africa. WHO | Regional Office for Africa. 2020 [cited 2022 Jan 5]. Available from: https://www.afro.who.int/news/new-covid-19-rapid-tests-game-changer-africa.

[103] Wiens KE, Mawien PN, Rumunu J, Slater D, Jones FK, Moheed S, et al. Seroprevalence of severe acute respiratory syndrome coronavirus 2 IgG in Juba, South Sudan, 2020 - Volume 27, Number 6—June 2021 - Emerging Infectious Diseases journal - CDC. 2021 [cited 2021 Jul 24]; Available from: https://wwwnc.cdc.gov/eid/article/27/6/21-0568_article. 10.3201/eid2706.210568 PMC8153877 34013872

[104] IASC. Public health and social measures for COVID-19 preparedness and response in low capacity and humanitarian settings. 2020 [cited 2021 Dec 22]. Available from: https://www.who.int/publications/m/item/public-health-and-social-measures-for-covid-19-preparedness-and-response-in-low-capacity-and-humanitarian-settings.

[105] WHO. Critical preparedness, readiness and response actions for COVID-19. 2021 [cited 2021 Dec 13]. Available from: https://www.who.int/emergencies/diseases/novel-coronavirus-2019/technical-guidance-publications.

[106] Dahab M, van Zandvoort K, Flasche S, Warsame A, Ratnayake R, Favas C, et al. COVID-19 control in low-income settings and displaced populations: what can realistically be done? Confl Health. 2020;14(1):54. Available from: 10.1186/s13031-020-00296-8. 10.1186/s13031-020-00296-8 PMC7393328 32754225

[107] WHO South Sudan. COVID-19 Update for South Sudan - 23 December 2021 - South Sudan. ReliefWeb. 2021 [cited 2022 Jan 5]. Available from: https://reliefweb.int/report/south-sudan/covid-19-update-south-sudan-23-december-2021.

[108] South Sudan Ministry of Health. South Sudan COVID-19 National Deployment and Vaccination Plan (Updated version 20th August 2021) - South Sudan. ReliefWeb. 2021 [cited 2021 Dec 22]. Available from: https://reliefweb.int/report/south-sudan/south-sudan-covid-19-national-deployment-and-vaccination-plan-updated-version.

[109] WHO. Less than 10% of African countries to hit key COVID-19 vaccination goal. WHO | Regional Office for Africa. 2021 [cited 2021 Dec 22]. Available from: https://www.afro.who.int/news/less-10-african-countries-hit-key-covid-19-vaccination-goal.

[110] WHO. Country support missions to help drive optimal COVID-19 vaccine uptake across Africa. WHO | Regional Office for Africa. 2021 [cited 2022 Jan 5]. Available from: https://www.afro.who.int/news/country-support-missions-help-drive-optimal-covid-19-vaccine-uptake-across-africa.

[111] Wrapp D, Wang N, Corbett KS, Goldsmith JA, Hsieh C-L, Abiona O (2020). Cryo-EM structure of the 2019-nCoV spike in the prefusion conformation. Science..

[112] Boyton RJ, Altmann DM (2021). The immunology of asymptomatic SARS-CoV-2 infection: what are the key questions?. Nat Rev Immunol..

[113] Huang C, Wang Y, Li X, Ren L, Zhao J, Hu Y (2020). Clinical features of patients infected with 2019 novel coronavirus in Wuhan, China. Lancet..

[114] Lin L, Lu L, Cao W, Li T (2020). Hypothesis for potential pathogenesis of SARS-CoV-2 infection–a review of immune changes in patients with viral pneumonia. Emerg Microbes Infect..

[115] Ragab D, Salah Eldin H, Taeimah M, Khattab R, Salem R (2020). The COVID-19 cytokine storm; what we know so far. Front Immunol..

[116] Mi J, Zhong W, Huang C, Zhang W, Tan L, Ding L (2020). Gender, age and comorbidities as the main prognostic factors in patients with COVID-19 pneumonia. Am J Transl Res..

[117] Yancy CW (2020). COVID-19 and African Americans. JAMA..

[118] Selden TM, Berdahl TA (2020). COVID-19 and racial/ethnic disparities in health risk, Employment, And Household Composition. Health Aff (Millwood)..

[119] Kusi KA, Frimpong A, Partey FD, Lamptey H, Amoah LE, Ofori MF (2021). High infectious disease burden as a basis for the observed high frequency of asymptomatic SARS-CoV-2 infections in sub-Saharan Africa. AAS Open Res..

[120] Velikova TV, Kotsev SV, Georgiev DS, Batselova HM (2020). Immunological aspects of COVID-19: what do we know?. World J Biol Chem..

[121] Smits VAJ, Hernández-Carralero E, Paz-Cabrera MC, Cabrera E, Hernández-Reyes Y, Hernández-Fernaud JR (2021). The Nucleocapsid protein triggers the main humoral immune response in COVID-19 patients. Biochem Biophys Res Commun..

[122] Xiaojie S, Yu L, Lei Y, Guang Y, Min Q. Neutralizing antibodies targeting SARS-CoV-2 spike protein. Stem Cell Research. 2021;50:102125. 10.1016/j.scr.2020.102125 PMC7737530 33341604

[123] Pušnik J, Richter E, Schulte B, Dolscheid-Pommerich R, Bode C, Putensen C (2021). Memory B cells targeting SARS-CoV-2 spike protein and their dependence on CD4+ T cell help. Cell Reports..

[124] Tso FY, Lidenge SJ, Peña PB, Clegg AA, Ngowi JR, Mwaiselage J (2021). High prevalence of pre-existing serological cross-reactivity against severe acute respiratory syndrome coronavirus-2 (SARS-CoV-2) in sub-Saharan Africa. Int J Infect Dis..

[125] Klompus S, Leviatan S, Vogl T, Kalka I, Godneva A, Shinar E, et al. Cross-reactive antibody responses against SARS-CoV-2 and seasonal common cold coronaviruses. medRxiv. 2020;2020.09.01.20182220.

[126] McCallum M, Walls AC, Sprouse KR, Bowen JE, Rosen LE, Dang HV, et al. Molecular basis of immune evasion by the Delta and Kappa SARS-CoV-2 variants. Science. 0:eabl8506. 10.1126/science.abl8506 PMC12240541 34751595

[127] Cohen KW, Linderman SL, Moodie Z, Czartoski J, Lai L, Mantus G (2021). Longitudinal analysis shows durable and broad immune memory after SARS-CoV-2 infection with persisting antibody responses and memory B and T cells. Cell Reports Medicine..

[128] Mateus J, Grifoni A, Tarke A, Sidney J, Ramirez SI, Dan JM, et al. Selective and cross-reactive SARS-CoV-2 T cell epitopes in unexposed humans. Science. 2020; 10.1126/science.abd3871. 10.1126/science.abd3871 PMC7574914 32753554

[129] Grifoni A, Sidney J, Zhang Y, Scheuermann RH, Peters B, Sette A. A sequence homology and bioinformatic approach can predict candidate targets for immune responses to SARS-CoV-2. Cell Host Microbe. 2020;27:671-680.e2. 10.1016/j.chom.2020.03.002 PMC7142693 32183941

[130] Chisale MRO, Ramazanu S, Mwale SE, Kumwenda P, Chipeta M, Kaminga AC, et al. Seroprevalence of anti-SARS-CoV-2 antibodies in Africa: a systematic review and meta-analysis. Rev Med Virol. 2021;:e2271. 10.1002/rmv.2271 PMC8420234 34228851

[131] Nwosu K, Fokam J, Wanda F, Mama L, Orel E, Ray N (2021). SARS-CoV-2 antibody seroprevalence and associated risk factors in an urban district in Cameroon. Nat Commun..

[132] Quashie PK, Mutungi JK, Dzabeng F, Oduro-Mensah D, Opurum PC, Tapela K (2021). Trends of SARS-CoV-2 antibody prevalence in selected regions across Ghana.

[133] Etyang AO, Lucinde R, Karanja H, Kalu C, Mugo D, Nyagwange J, et al. Seroprevalence of antibodies to severe acute respiratory syndrome coronavirus 2 among healthcare workers in Kenya. Clin Infect Dis. 2021;:ciab346. 10.1093/cid/ciab346 PMC8135298 33893491

[134] Uyoga S, Adetifa IMO, Karanja HK, Nyagwange J, Tuju J, Wanjiku P (2021). Seroprevalence of anti-SARS-CoV-2 IgG antibodies in Kenyan blood donors. Science..

[135] Mandolo J, Msefula J, Henrion MYR, Brown C, Moyo B, Samon A (2021). SARS-CoV-2 exposure in Malawian blood donors: an analysis of seroprevalence and variant dynamics between January 2020 and July 2021. BMC Medicine..

[136] Woudenberg T, Pelleau S, Anna F, Attia M, Donnadieu F, Gravet A (2021). Humoral immunity to SARS-CoV-2 and seasonal coronaviruses in children and adults in north-eastern France. EBioMedicine..

[137] Lee CH, Pinho MP, Buckley PR, Woodhouse IB, Ogg G, Simmons A (2020). Potential CD8+ T cell cross-reactivity against SARS-CoV-2 conferred by other coronavirus strains. Front Immunol..

[138] Grifoni A, Weiskopf D, Ramirez SI, Mateus J, Dan JM, Moderbacher CR, et al. Targets of T cell responses to SARS-CoV-2 coronavirus in humans with COVID-19 disease and unexposed individuals. Cell. 2020;:S0092867420306103. 10.1016/j.cell.2020.05.015 PMC7237901 32473127

[139] Dobaño C, Santano R, Jiménez A, Vidal M, Chi J, Melero NR (2020). Immunogenicity and crossreactivity of antibodies to SARS-CoV-2 nucleocapsid protein.

[140] Boyce MR, Katz R, Standley CJ (2019). Risk factors for infectious diseases in urban environments of Sub-Saharan Africa: a systematic review and critical appraisal of evidence. Trop Med Infect Dis..

[141] Ademolue TW, Aniweh Y, Kusi KA, Awandare GA. Patterns of inflammatory responses and parasite tolerance vary with malaria transmission intensity. Malar J. 2017;16. 10.1186/s12936-017-1796-x PMC5387356 28399920

[142] Wamae K, Wambua J, Nyangweso G, Mwambingu G, Osier F, Ndung’u F (2019). Transmission and age impact the risk of developing febrile malaria in children with asymptomatic Plasmodium falciparum Parasitemia. J Infect Dis..

[143] Hong M, Bertoletti A (2017). Tolerance and immunity to pathogens in early life: insights from HBV infection. Semin Immunopathol..

[144] Vitetta L, Vitetta G, Hall S (2018). Immunological tolerance and function: associations between intestinal bacteria, probiotics, prebiotics, and phages. Front Immunol..

[145] Yap GS, Gause WC. Helminth infections induce tissue tolerance mitigating immunopathology but enhancing microbial pathogen susceptibility. Front Immunol. 2018;9. 10.3389/fimmu.2018.02135 PMC6198046 30386324

[146] Bickett TE, McLean J, Creissen E, Izzo L, Hagan C, Izzo AJ, et al. Characterizing the BCG induced macrophage and neutrophil mechanisms for defense against Mycobacterium tuberculosis. Front Immunol. 2020;11. 10.3389/fimmu.2020.01202 PMC7314953 32625209

[147] Miller A, Reandelar MJ, Fasciglione K, Roumenova V, Li Y, Otazu G (2020). Correlation between universal BCG vaccination policy and reduced mortality for COVID-1.

[148] Yengil E, Onlen Y, Ozer C, Hambolat M, Ozdogan M (2021). Effectiveness of booster measles-mumps-rubella vaccination in lower COVID-19 infection rates: a retrospective cohort study in Turkish adults. IJGM..

[149] Malave Sanchez M, Saleeb P, Kottilil S, Mathur P. Oral polio vaccine to protect against COVID-19: out of the box strategies? Open Forum Infect Dis. 2021;8:ofab367. 10.1093/ofid/ofab367 PMC8344522 34381846

[150] Global Change Data Lab. Cumulative confirmed COVID-19 cases by world region. Our World in Data. 2022 [cited 2022 Feb 8]. Available from: https://ourworldindata.org/grapher/cumulative-covid-cases-region.

[151] Africa CDC. Africa identifies first case of coronavirus disease: statement by the Director of Africa CDC – Africa CDC. [cited 2022 Feb 8]. Available from: https://africacdc.org/news-item/africa-identifies-first-case-of-coronavirus-disease-statement-by-the-director-of-africa-cdc/.

[152] WHO. WHO issues its first emergency use validation for a COVID-19 vaccine and emphasizes need for equitable global access. Who. 2020 [cited 2022 Feb 8]. p. 2020–2. Available from: https://www.who.int/news/item/31-12-2020-who-issues-its-first-emergency-use-validation-for-a-covid-19-vaccine-and-emphasizes-need-for-equitable-global-access.

[153] Gavi. COVAX explained | Gavi, the Vaccine Alliance. Website. 2021 [cited 2022 Feb 17]. p. 1–1. Available from: https://www.gavi.org/vaccineswork/covax-explained.

[154] WHO. WHO lists two additional COVID-19 vaccines for emergency use and COVAX roll-out. WHO.int. 2021 [cited 2022 Feb 17]. p. 19–21. Available from: https://www.who.int/news/item/15-02-2021-who-lists-two-additional-covid-19-vaccines-for-emergency-use-and-covax-roll-out

[155] Tavilani A, Abbasi E, Ara FK, Darini A, Asefy Z. COVID-19 vaccines: current evidence and considerations. Metab Open. 2021 [cited 2022 Feb 17];12:100124. Available from: https://www.ncbi.nlm.nih.gov/pmc/articles/PMC8433053/. 10.1016/j.metop.2021.100124 PMC8433053 34541483

[156] WHO. First COVID-19 COVAX vaccine doses administered in Africa. WHO News. 2021 [cited 2022 Feb 7]. Available from: https://www.who.int/news/item/01-03-2021-first-covid-19-covax-vaccine-doses-administered-in-africa.

[157] Mathieu E, Ritchie H, Ortiz-Ospina E, Roser M, Hasell J, Appel C, Giattino C, Rodés-Guirao L. Coronavirus pandemic (COVID-19). Our World Data. 2020;5(7):947–53. [cited 2022 Feb 13] Available from: https://ourworldindata.org/coronavirus.

[158] World Health Organization. Africa COVID-19 vaccination dashboard. 2022 [cited 2022 Feb 13]. Available from: https://app.powerbi.com/view?r=eyJrIjoiY2ViYzIyZjItYzhkMi00ZWVkLTgyM2ItZTk1ZTJmODRjMTkxIiwidCI6ImY2MTBjMGI3LWJkMjQtNGIzOS04MTBiLTNkYzI4MGFmYjU5MCIsImMiOjh9.

[159] Coronavirus (COVID-19) vaccinations - our world in data. [cited 2022 Feb 13]. Available from: https://ourworldindata.org/covid-vaccinations.

[160] World Health Organization. WHO, UN set out steps to meet world COVID vaccination targets. World Health Organization. 2021 [cited 2022 Feb 13]. Available from: https://www.who.int/news/item/07-10-2021-who-un-set-out-steps-to-meet-world-covid-vaccination-targets.

[161] Mwai P. Covid-19 vaccinations: African nations miss WHO target - BBC News. BBC Reality Check. 2021 [cited 2022 Feb 13]. Available from: https://www.bbc.com/news/56100076.

[162] Mwai P. Covid in Tanzania: vaccination campaign gets underway - BBC News. BBC Reality Check. 2021 [cited 2022 Feb 13]. Available from: https://www.bbc.com/news/57641824.

[163] Burundi launches COVID-19 vaccination drive | Reuters. [cited 2022 Feb 13]. Available from: https://www.reuters.com/world/africa/burundi-launches-covid-19-vaccination-drive-2021-10-18/.

[164] Torreele E, Amon JJ. Equitable COVID-19 vaccine access. Health Hum Rights. 2021;23(1):273. [cited 2022 Feb 16]. Available from: https://www.ncbi.nlm.nih.gov/pmc/articles/PMC8233010/. PMC8233010 34194219

[165] Ten steps to prepare for COVID-19 vaccine rollout in Africa | WHO | Regional Office for Africa. [cited 2022 Feb 13]. Available from: https://www.afro.who.int/news/ten-steps-prepare-covid-19-vaccine-rollout-africa.

[166] Africa CDC. Implementation Guide for COVID-19 Vaccines in Africa. 2020 [cited 2022 Feb 13]. Available from: https://africacdc.org/download/implementation-guide-for-covid-19-vaccines-in-africa/.

[167] McAdams D, McDade KK, Ogbuoji O, Johnson M, Dixit S, Yamey G (2020). Incentivising wealthy nations to participate in the COVID-19 Vaccine Global Access Facility (COVAX): a game theory perspective. BMJ Glob Heal..

[168] Li Z, Lu J, Lv J. The inefficient and unjust global distribution of COVID-19 vaccines: from a perspective of critical global justice. Inq (United States). 2021;58:1–8. 10.1177/00469580211060992 PMC8649905 34865544

[169] Binagwaho A, Mathewos K, Davis S. Equitable and effective distribution of the COVID-19 vaccines-a scientific and moral obligation editorial. Int J Heal Policy Manag. 2021;2021:1–3. Available from: http://ijhpm.com. 10.34172/ijhpm.2021.49 PMC9278601 33949818

[170] Feinmann J. Covid-19: global vaccine production is a mess and shortages are down to more than just hoarding. BMJ. 2021;375. [cited 2022 Feb 13] Available from: https://www.bmj.com/content/375/bmj.n2375. 10.1136/bmj.n2375 34711605

[171] Stein F (2021). Risky business: COVAX and the financialization of global vaccine equity. Global Health..

[172] World Health Organization. Joint Statement on Dose Donations of COVID-19 Vaccines to African Countries. Africa CDC. 2021 [cited 2022 Feb 16]. Available from: https://www.who.int/news/item/29-11-2021-joint-statement-on-dose-donations-of-covid-19-vaccines-to-african-countries.

[173] COVAX. COVID-19 vaccine introduction toolkit. World Health Organization. 2021 [cited 2022 Feb 16]. Available from: https://www.who.int/tools/covid-19-vaccine-introduction-toolkit.

[174] Arevshatian L, Clements C, Lwanga S, Misore A, Ndumbe P, Seward J, Taylor P. An evaluation of infant immunization in Africa: is a transformation in progress? Bull World Health Organ. 2007;85(6):449–57. [cited 2022 Feb 16]. Available from: http://www.who. 10.2471/BLT.06.031526 PMC2636339 17639242

[175] UN. World Population Prospects - Population Division. World Population Prospects - 2015 Revision. 2019 [cited 2022 Mar 8]. Available from: https://population.un.org/wpp/Graphs/Probabilistic/POP/TOT/231.

[176] Nature. Africa is bringing vaccine manufacturing home. Nature. 2022;602(7896):184. [cited 2022 Feb 16]. Available from: https://www.nature.com/articles/d41586-022-00335-9. 10.1038/d41586-022-00335-9 35140392

[177] Bloomberg. BioNTech to send Covid vaccine production units to Africa - Bloomberg. 2022 [cited 2022 Mar 8]. Available from: https://www.bloomberg.com/news/articles/2022-02-16/biontech-to-send-covid-vaccine-production-units-to-africa.

[178] Mboussou F, Ndumbi P, Ngom R, Kamassali Z, Ogundiran O, Van Beek J, Williams G, Okot C, Hamblion EL, Impouma B (2019). Infectious disease outbreaks in the African region: overview of events reported to the World Health Organization in 2018. Epidemiol Infect..

[179] Nyakarahuka L, Ayebare S, Mosomtai G, Kankya C, Lutwama J, Mwiine FN, et al. Ecological niche modeling for filoviruses: a risk map for Ebola and Marburg virus disease outbreaks in Uganda. PLoS currents. 2017;9. 10.1371/currents.outbreaks.07992a87522e1f229c7cb023270a2af1 PMC5614672 29034123

[180] Labbé J, Ford JD, Berrang-Ford L, Donnelly B, Lwasa S, Namanya DB, Twesigomwe S, Harper SL, Team IR (2016). Vulnerability to the health effects of climate variability in rural southwestern Uganda. Mitig Adapt Strateg Glob Chang..

[181] Riley T, Sully E, Ahmed Z, Biddlecom A (2020). Estimates of the potential impact of the COVID-19 pandemic on sexual and reproductive health in low- and middle-income countries. Int Perspect Sex Reprod Health..

[182] Muoki. Impact assessment of COVID-19 pandemic on the tourism and hospitality industry in the EAC and post recovery strategy for the sector. AERC Working Paper - COVID-19_018 African Economic Research Consortium, Nairobi September 2021. World Health Organization 2021. Maintaining the provision and use of services for maternal, newborn, child and adolescent health and older people during the COVID-19 pandemic: lessons learned from 19 countries. ISBN 978-92-4-004059-5 (electronic version). ISBN 978-92-4-004060-1 (print version).

[183] Jabeen S (2016). Do we really care about unintended outcomes? An analysis of evaluation theory and practice. Eval Progr Plan.

[184] Turcotte-Tremblay A-M, Gali IAG, Ridde V (2021). The unintended consequences of COVID-19 mitigation measures matter: practical guidance for investigating them. BMC Med Res Methodol.

[185] Chi Y-L, Regan L, Nemzoff C, Krubiner C, Anwar Y, Walker D (2020). Beyond COVID-19: a whole of health look at impacts during the pandemic response.

[186] Colebunders R, Fodjo JNS, Vanham G, Van den Bergh R (2020). A call for strengthened evidence on targeted, non-pharmaceutical interventions against COVID-19 for the protection of vulnerable individuals in sub-Saharan Africa. Int J Infect Dis.

[187] Nghochuzie NN, Olwal CO, Udoakang AJ, Amenga-Etego LN-K, Amambua-Ngwa A (2020). Pausing the fight against malaria to combat the COVID-19 pandemic in Africa: is the future of malaria bleak?. Front Microbiol.

[188] Karamagi HC, Tumusiime P, Titi-Ofei R, Droti B, Kipruto HK, Nabyonga-Orem J, Binetou-Wahebine Seydi A, Zawaira F, Schmets G, Cabore JW (2021). Towards universal health coverage in the WHO African Region: assessing health system functionality, incorporating lessons from COVID-19. BMJ Glob Health..

[189] Karamagi HC, Titi-Ofei R, Kipruto HK, Binetou-Wahebine Seydi, Talisuna A, Tsofa B, Saikat S, Schmets G, Barasa E, Tumusiime P, Makubalo L, Cabore JW, Moeti M. On the resilience of health systems: a methodological exploration across countries in the WHO African Region. PLoS One. 2022;17(2):e0261904. 10.1371/journal.pone.0261904 PMC8820618 35130289

[190] UNDP. The impact of the Covid-19 pandemic in Nigeria: a socio-economic analysis. UNDP Brief; 2020.

[191] Gupta M, Wahl B, Adhikari B, Bar-Zeev N, Bhandari S, Coria A, Erchick DJ, Gupta N, Hariyani S, Kagucia EW, Killewo J, Limaye RJ, McCollum ED, Pandey R, Pomat WS, Rao KD, Santosham M, Sauer M, Wanyenze RK, Peters DH (2020). The need for COVID-19 research in low- and middle-income countries. Glob Health Res Policy..

[192] Younger SD, Musisi A, Asiimwe W, Ntungire N, Rauschendorfer J, Manwaring P: Estimating income losses and consequences of the COVID-19 crisis in Uganda. In.: Tulane University, Department of Economics; 2020. Knowledge is Wealth: Helping Coffee Farmers Face the COVID-19 Crisis. https://www.technoserve.org/blog/knowledge-is-wealth-helping-coffee-farmers-face-the-covid-19-crisis/.

[193] Kabwama S, Kiwanuka SN, Monje F, Ndejjo R, Kizito S, Wanyenze RK. Essential Health Services: Uganda; Exemplars in Global Health. https://www.exemplars.health/emerging-topics/epidemic-preparedness-and-response/essential-health-services/uganda.

[194] Oladimeji O, Atiba BP, Adeyinka DA. Leveraging polymerase chain reaction technique (GeneXpert) to upscaling testing capacity for SARS-CoV-2 (COVID-19) in Nigeria: a game changer. Pan Afr Med J. 2020;35(Suppl 2):8. 10.11604/pamj.2020.35.2.22693. eCollection 2020 10.11604/pamj.2020.35.2.22693 PMC7268909 32528619

[195] Exemplars in Global Health. Overview Essential Health Services. https://www.exemplars.health/emerging-topics/epidemic-preparedness-and-response/essential-health-services.

[196] Shapira G, Ahmed T, Drouard SHP, Amor Fernandez P, Kandpal E, Nzelu C, et al. Disruptions in maternal and child health service utilization during COVID-19: analysis from eight sub-Saharan African countries. Health Policy Plan. 2021;36(7):1140–51. 10.18593/heapol/czab064. 10.1093/heapol/czab064 PMC8344431 34146394

[197] COVID-19 pandemic cuts access to sexual and reproductive healthcare for women around the world. https://www.ippf.org/news/covid-19-pandemic-cuts-access-sexual-and-reproductive-healthcare-women-around-world.

[198] World Health Organization (WHO). Coronavirus disease (COVID-19) Weekly Epidemiological Update and Weekly Operational Update. https://www.who.int/emergencies/diseases/novel-coronavirus-2019/situation-reports. Accessed 14 Fen 2022.

[199] Murewanhema G, Makurumidze R. Essential health services delivery in Zimbabwe during the COVID-19 pandemic: perspectives and recommendations. Pan Afr Med J. 2020;35(Suppl 2):143. doi: 10.11604/pamj.supp.2020.35.143.25367. 10.11604/pamj.supp.2020.35.143.25367 PMC7608772 33193958

[200] Desta AA, Woldearegay TW, Gebremeskel E, Alemayehu M, Getachew T, Gebregzabiher G, Ghebremedhin KD, Zgita DN, Aregawi AB, Redae G (2021). Impacts of COVID-19 on essential health services in Tigray, Northern Ethiopia: a pre-post study. PLoS One..

[201] Jensen C, McKerrow NH. Child health services during a COVID-19 outbreak in KwaZulu-Natal Province, South Africa, S Afr Med J. 2020;0(0):13185. 33334393

[202] Hakizimana D, Ntizimira C, Mbituyumuremyi A, Hakizimana E, Mahmoud H, Birindabagabo P, Musanabaganwa C, Gashumba D (2022). The impact of Covid-19 on malaria services in three high endemic districts in Rwanda: a mixed-method study. Malar J..

[203] Barasa E, Kazungu J, Orangi S, Kabia E, Ogero M, Kasera K. Indirect health effects of the COVID-19 pandemic in Kenya: a mixed methods assessment. 10.1186/s12913-021-06726-4 PMC8311400 34311716

[204] Karp C, Wood SN, Guiella G, Gichangi P, Bell SO, Anglewicz P, Larson E, Zimmerman L, Moreau C (2021). Contraceptive dynamics during COVID-19 in sub-Saharan Africa: longitudinal evidence from Burkina Faso and Kenya. BMJ Sex Reprod Health..

[205] Nuwematsiko R, Nabiryo M, Bomboka JB, Nalinya S, Musoke D, Okello D, Wanyenze RK (2022). Unintended socio-economic and health consequences of COVID-19 among slum dwellers in Kampala, Uganda. BMC Public Health..

[206] Shem Okore Bodo, ADEA. COVID-19 and schools reopening in African countries: Twists and turns. 2021. https://www.globalpartnership.org/blog/covid-19-and-schools-reopening-african-countries-twists-and-turns.

[207] ADEA, AU/CIEFFA, APHRC. School reopening in Africa during the COVID-19 pandemic. Abidjan, Ouagadougou, Nairobi: ADEA, AU/CIEFFA, APHRC. 2021. https://www.adeanet.org/sites/default/files/school_reopening_kix_observatory.pdf.

[208] UNDP. Maximizing tourism’s contribution to Africa’s COVID-19 recovery. 2021. https://www.undp.org/press-releases/maximizing-tourisms-contribution-africas-covid-19-recovery.

[209] Africa Development Bank Group. 2021. Democratic Republic of Congo Economic Outlook. https://www.afdb.org/en/countries-central-africa-democratic-republic-congo/democratic-republic-congo-economic-outlook.

[210] World Bank: Monitoring social and economic impacts of COVID-19 on refugees in Uganda: results from the high-frequency phone survey-second round. In: World Bank; 2021.

[211] UNDP. Socio-economic impact of COVID-19 in Uganda: short-, medium-, and long-term impacts on poverty dynamics and SDGs using scenario anlaysis and system dynamics modeling: UNDP; 2020.

[212] Le Nestour A, Mbaye S, Moscoviz L: Enquête téléphonique sur la crise du Covid au Sénégal. Center for Global Development 2020:25.

[213] The Conversation. How COVID gave African countries the opportunity to improve public health. 2022. https://theconversation.com/how-covid-gave-african-countries-the-opportunity-to-improve-public-health-173335.

[214] African Union. Public-Private Partnership a viable path for Africa’s economic recovery; African Private Sector Forum. 2021. https://au.int/es/node/41162.

[215] Oxfam. The Ignored Pandemic: the dual crisis of gender-based violence and COVID-19. https://policy-practice.oxfam.org/resources/the-ignored-pandemic-the-dual-crises-of-gender-based-violence-and-covid-19-621309/.

[216] Apondi R, Awor A, Nelson L, Cheptoris J, Ngabirano F, Egbulem CD (2021). Gender-based violence shadows COVID-19: increased sexual violence, HIV exposure and teen pregnancy among girls and women in Uganda [conference abstract]. J Int AIDS Soc..

[217] HRW. Impact of Covid-19 on Children’s Education in Africa. 2020. https://www.hrw.org/news/2020/08/26/impact-covid-19-childrens-education-africa.

[218] UNESCO. Youth as researchers knowledge sharing: exploring the impact of COVID-19 in learning in Africa. 2021. https://en.unesco.org/news/exploring-impact-covid-19-learning-africa.

[219] WHO. Strengthening schools to ensure safe continuity of education amidst COVID-19. 2021. https://www.afro.who.int/news/strengthening-schools-ensure-safe-continuity-education-amidst-covid-19.

[220] UNESCO. Dashboards on the global monitoring of school closures caused by the COVID-19 Pan. https://covid19.uis.unesco.org/global-monitoring-school-closures-covid19/.

